# A Comprehensive Review of Self-Healing Polymer, Metal, and Ceramic Matrix Composites and Their Modeling Aspects for Aerospace Applications

**DOI:** 10.3390/ma15238521

**Published:** 2022-11-29

**Authors:** Sri Ram Murthy Paladugu, P. S. Rama Sreekanth, Santosh Kumar Sahu, K. Naresh, S. Arun Karthick, N. Venkateshwaran, Monsuru Ramoni, Rhoda Afriyie Mensah, Oisik Das, Ragavanantham Shanmugam

**Affiliations:** 1 School of Mechanical Engineering, VIT-AP University, Amaravati 522337, India; 2 Department of Chemical Engineering and Materials Science, University of Southern California, Los Angeles, CA 90089, USA; 3 Feynman Nano Laboratory, Department of Biomedical Engineering, Sri Sivasubramaniya Nadar College of Engineering, Chennai 603110, India; 4 Department of Mechanical Engineering, Rajalakshmi Engineering College, Chennai 600125, India; 5 School of Engineering, Math and Technology, Navajo Technical University, Crownpoint, NM 87313, USA; 6 Department of Civil, Environmental and Natural Resources Engineering, Lulea University of Technology, 97187 Lulea, Sweden

**Keywords:** microcapsules, hollow fibers, vascular network, healing mechanisms

## Abstract

Composites can be divided into three groups based on their matrix materials, namely polymer, metal and ceramic. Composite materials fail due to micro cracks. Repairing is complex and almost impossible if cracks appear on the surface and interior, which minimizes reliability and material life. In order to save the material from failure and prolong its lifetime without compromising mechanical properties, self-healing is one of the emerging and best techniques. The studies to address the advantages and challenges of self-healing properties of different matrix materials are very limited; however, this review addresses all three different groups of composites. Self-healing composites are fabricated to heal cracks, prevent any obstructed failure, and improve the lifetime of structures. They can self-diagnose their structure after being affected by external forces and repair damages and cracks to a certain degree. This review aims to provide information on the recent developments and prospects of self-healing composites and their applications in various fields such as aerospace, automobiles etc. Fabrication and characterization techniques as well as intrinsic and extrinsic self-healing techniques are discussed based on the latest achievements, including microcapsule embedment, fibers embedment, and vascular networks self-healing.

## 1. Introduction

Materials with self-healing capability have gained much attention recently. For example, scratches on a car can be patched or repaired on their own, restoring its original shiny appearance. This is somewhat similar to wound healing in mammals [1]. Structural materials deteriorate and degrade over time leading to micro-cracks that cause failure. Accordingly, to improve the reliability and lifetime of products, repair is necessary [2]. However, it is quite challenging to integrate the healing process of extreme conditions such as fractured bones into manufacturing products due to changing trends in the healing mechanisms of human bodies and other living beings [3]. 

Self-healing composites are capable of auto-repairing upon crack initiation and regaining their mechanical properties without disturbing the mode of application. Self-healing mechanisms can be divided into two types, extrinsic and intrinsic healing. In extrinsic healing, the healing agent is used as an additive to fill up the cracks in the matrix and in intrinsic healing, a reversible crosslinking bond (supramolecular chemistry) is used to bind the monomers and fill the cracks [4]. Furthermore, classification can also be made on autonomic healing or non-autonomic healing (i.e., with or without external stimuli). A few well-known methods for developing self-healing composites are the inclusion of microcapsules, hollow fibers, and a vascular network containing healing agents. Self-healing can also be achieved by thermal activation of reversible interactions or dissolved thermoplastic polymers [5].

The self-healing concept is an emerging technique in the engineering field and it can be applied to composite materials such as polymer matrix composites (PMCs), ceramic matrix composites (CMCs), metal matrix composites (MMCs), and cementitious composites. Apart from the specified areas, the self-healing concept can also be applied to coatings for corrosion protection for commercial applications [6,7]. Many potential applications of self-healing composites are implemented in resistant fabrics, resealing tires, long-life batteries, aerospace sectors, and automobile fields. In the aerospace sector, the damage due to impact load is severe [8]. However, self-healing materials can repair damage caused due to outside environment and increase the lifetime of the components. One key application of self-healing materials is to repair dynamic damage and maintain impact resistance [9].

**Figure 1:**
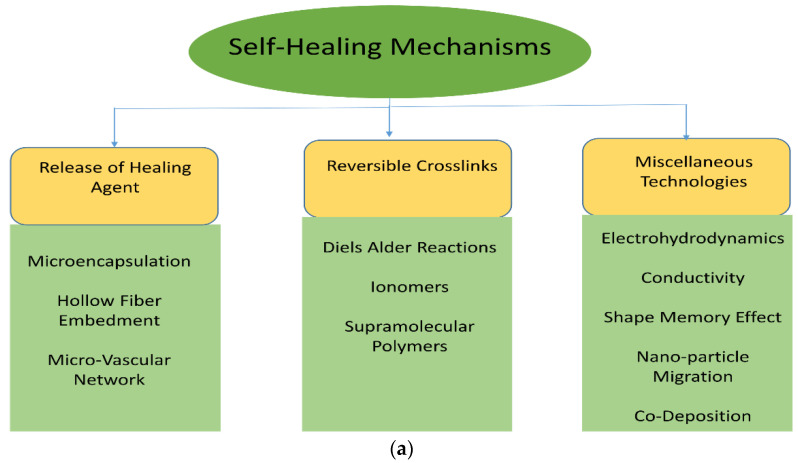

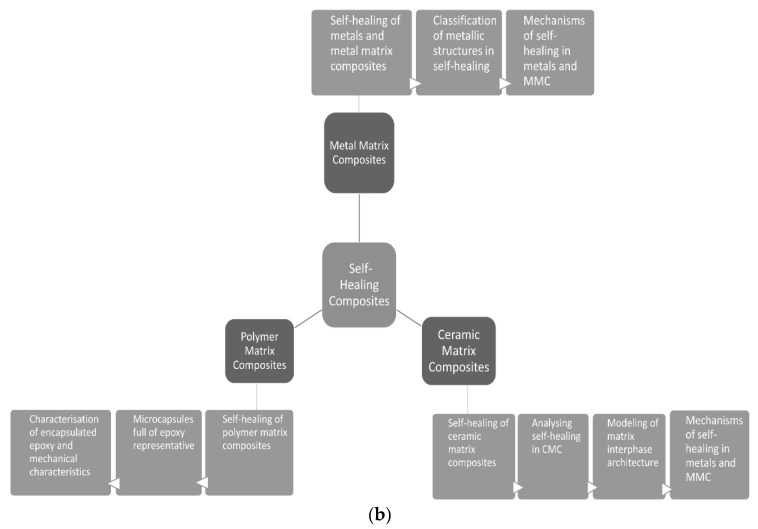
Schematic representation of concepts covered in the article. (**a**) Overview of Self-healing mechanisms and chemistry. (**b**) Applying self-healing techniques in various composites such as metals, polymers, and ceramics.

This study aims to understand the self-healing mechanisms (Figure 1) and their techniques which can be applied in day-to-day applications and in emerging fields such as structures, aerospace, automobiles, etc. to improve the life span, and working time and reduce the cost of manufacturing the materials by repairing the existing materials and increase the reliability of materials. Initially, self-healing mechanisms such as microcapsules, hollow fiber embedment, vascular network, and intrinsic healing are explained in subsequent sections. This study also aims to understand the chemistry behind the process of self-healing and implement them in various composites such as polymer matrix composites, metal matrix composites, and ceramic matrix composites to improve their mechanical properties after damage and increase the healing efficiency explained clearly. Finally, self-healing composites in aerospace applications were discussed.

### 1.1. Principle of Self-Healing

The capacity of a material to heal (recover/repair) from damages naturally and autonomously without any external or superficial interference is described as self-healing. Several common words, such as self-repair, autonomic healing, and automatic repair, are implemented in materials to describe their characteristics. Some products also require external intervention to initiate self-healing characteristics [4,5,6]. Thus, there are two modes of self-healing processes: autonomic (no necessary external intervention) and non-autonomic (needs human triggering or external intervention).

Numerous forms of healing mechanisms are treated in this analysis as, in general, self-healing. The development of cracks in materials reduces the mechanical properties, hence self-healing can be used to recover mechanical characteristics by healing the cracks. Nevertheless, it has been reported that filling and restoring even small pinholes irrespective of cracks is possible to improve the mechanical performance of materials [7,8,9,10].

#### 1.1.1. Design Strategies

Various material types, such as polymers or plastics, coatings or paints, alloys or metals, and concrete or cementitious alloys, have different systems for self-healing. In this review, various self-healing methods are explored. Strategies have been established with specificity based on different materials and their corresponding characteristics. The design prototype of self-healing components is discussed below:

- Release of healing agent
- Reversible crosslinks
- Miscellaneous technologies such as electro-hydrodynamics, conductivity, shape memory alloy, nanoparticle migration, co-deposition, etc. [11,12,13].

#### 1.1.2. Release of Healing Agent

Microencapsulation is a mechanism by which micron-sized solid particles or droplets of liquids are sealed in inert shell organs to separate and shield them from outside environments. The idleness is associated with the shell’s reactivity to the encapsulated material. The outcome obtained from the method of microencapsulation was known as microcapsules. To design self-healing polymer composites, microcapsules with catalysts and healing agents are used. Table 1 shows the factors which are considered while developing microcapsule embedded in the matrix. Early research [14] shows that employing microencapsulated healings in a matrix of polyester to generate a self-healing action is advised. However, they proved ineffectual in attaining functioning self-healing goods. Maira et al. [15] performed the first experimental demonstration of self-healing materials by integrating healing agents via encapsulation into the polymer matrix comprising scattered catalysts. The authors used “DCPD” (dicyclopentadiene) as a chemical healing material and a liquid healing agent in their work. As an internal chemical stimulus, Grubb’s catalyst [bis (tricyclohexylphosphine) benzylidene ruthenium (IV) dichloride] and epoxy matrix were used for dispersion since the monomer has high durability, low viscosity, and is comparatively less costly [16,17,18,19,20].

**Figure 2:**
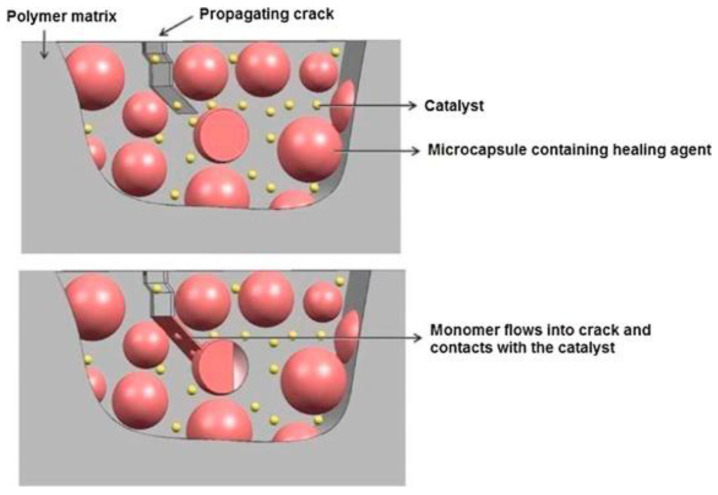

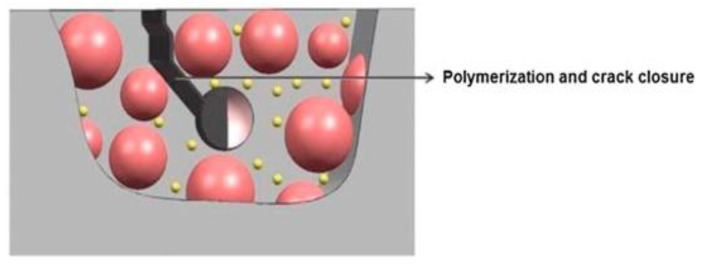
Self-healing process using microcapsules [21].

During the development process, liquid ingredients such as dyes, monomers, hardeners, catalysts dyes, and hollow channels are inserted into polymer-generated structures. These pools are ruptured in the event of a fracture, and the reactive agents are poured into fissures by capillary force, where they solidify in the presence of pre-dispersed catalysts to heal the crack (see Figure 2). The primary driver of this process is the formation of cracks. On the other hand, it is challenging to ease the tension from the fracture, which is a massive drawback of this procedure. However, this strategy needs no external input since it is independent. By using this notion of self-healing material design, several options were examined as follows.

#### 1.1.3. Microcapsule Embedment

**Figure 3:**
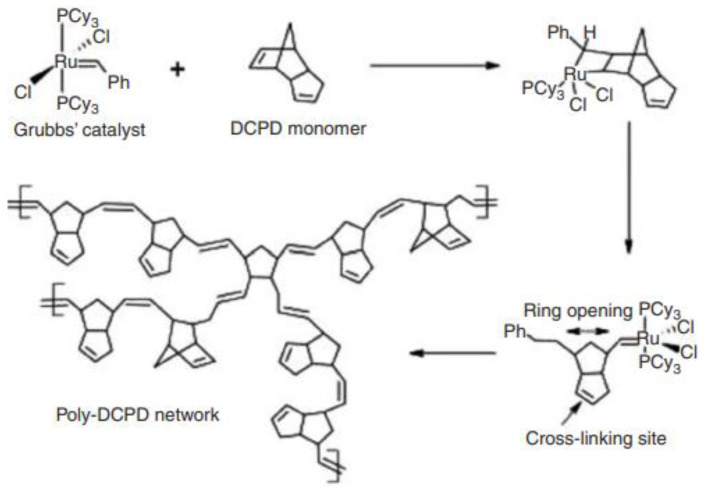
ROMP (Ring opening metathesis polymerization) of DCPD [6].

A ring-opening metathesis polymerization (ROMP) occurs, and strongly polymerized metathesis begins as Grubbs catalyst and Dicyclopentadiene (DCPD) are merged. The compounds come into contact when dispersed in an epoxy resin [22,23]. Tough polycyclopentadiene which is cross-linked can plug the crack as shown in Figure 3. Monomer which is low viscous allows it to move across the plane of fracture. According to the experts, fracture regeneration toughness may be increased by 75% compared to the basic specimen [24].

The addition of monomers such as hydroxyl-functionalized polydimethylsiloxane (HOPDMS) and polydiethylsiloxane (PDES) to a synthetic ester base matrix, persists as droplets in microphase separation. The writers utilized encapsulated catalyst instead of a contained monomer healing agent. Inside the matrix, microcapsules of polyurethane with the di-n-dibutyltin dilaurate (DBTL) catalyst are then eliminated [25,26,27,28,29]. The catalyst reacts with the monomer whenever these capsules burst, thus, polycondensation happens. Researchers created self-healing elastomers on polydimethylsiloxane (PDMS) by combining two types of micro-sized capsules: a resin and an initiator capsule. References [30,31,32] looked at the efficiency of tiny capsules in self-recovery. The authors recently posted the creation of self–healing polymer composites without catalysts. Numerous research is being conducted in this area and it is undoubtedly complex due to these discoveries. The authors recently demonstrated using latent curing, CuBr2 (2-Melm) 4, rather than a solid stage, to produce self-mending materials via ROMP reaction.

#### 1.1.4. Hollow Fiber Embedment

The self-healing solution based on microcapsules has the main drawback of instability in resulting in thorough and multiple healing since it consists of a small supply of healing agents. Multiple healing is only possible if there is the existence of overheating agent in the matrix after the initial healing. As a result, a new reservoir capable of delivering larger quantities of liquid healing agents to attain multiple healing in composite materials has been developed [33]. However, they have only had little success with their technique. Broad-diameter capillaries were later placed in resins [34], but the trials were unsuccessful. Smaller hollow glass fibers, also known as Hollex fibers, were placed in resins [35]. However, composites formulated based on glass-reinforced fibers failed to produce healing in the crack. Strong viscous resins were not suitable and curing was terrible.

**Figure 4:**
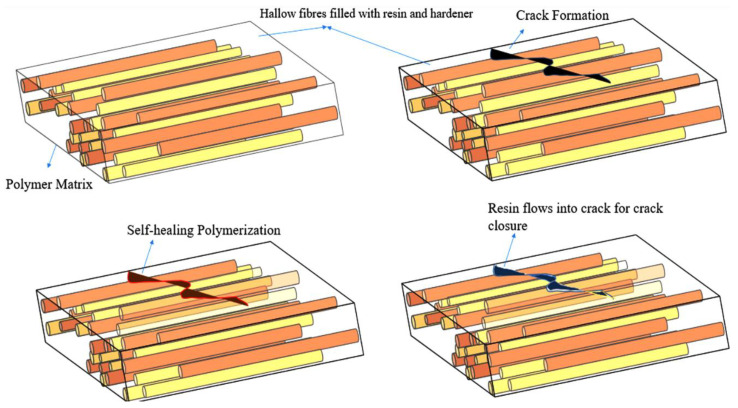
Self-healing of Hollow fibers [21].

Chen et al. [36] developed a method for optimizing the manufacturing of hollow fiber glasses to be used as a liquid curing agent and dye container. Borosilicate glass fiber has a diameter of 30 to 100 micrometers and a 55% hollow (Figure 4). They prepared hollow fibers using composite panels and recovered up to 97% of the original flexural power by containing repair agents [37,38,39,40,41,42]. Additionally, the authors reported the penetration of fluorescent dye along broken hollow fibers into the crack plane. Having these self-healing material construction techniques provides some benefits, which are as follows:

- To restore injury, a more considerable amount of curing agent should be available.
- Various activation methods/resin forms can be chosen.
- Visual examination of the affected region is possible.
- Hollow fibers can be quickly combined and adapted with traditional reinforcing fibers.

#### 1.1.5. Micro Vascular Systems

Another strategy analogous to the natural vascular system of several flowers and animals was investigated to resolve the restricted method of inserting a healing agent into the microcapsule [43,44]. This technology is dependent on a community with a continuously centralized administration of healing solutions to the polymer structure.

The production procedure is sophisticated for such networks for practical applications, and it is not easy to make synthetic materials. Chemical links are deposited in this phase following a 3D array, and an epoxy resin infiltrates the interstitial pores within the printed lines. The fugitive ink is extracted until the polymer is repaired, leaving only a 3D microvascular channel. Microvascular networked polymeric structures were developed by integrating catalysts in the polymer, which helps to penetrate the matrix. The curing agent is less effective in the microvascular channels when healing the polymer and extracting the scaffold. Several studies have documented such manufacturing techniques and their associated self-healing capacities [45].

From the above-mentioned extrinsic techniques, microcapsule embedment is widely used in the industry. Generally, microcapsules are made from urea formaldehyde and triethylenetetramine (TETA) microcapsules were used for wear-resistant polymer composite. Placing the healing agent and catalyst in the matrix can be followed in four ways; (1) embedding the healing agent and catalyst in single microcapsules. (2) Embedding healing agent and catalyst in different capsules and placing in the matrix and making sure they react with the external stimuli. (3) Placing healing agent in the microcapsules alone which reacts with the functionalization of the matrix. (4) Embedding healing agent or catalyst in different phases of matrix [40,41,42]. However, the microcapsule technique has a limitation as it is constrained to a single application to fill the crack in the matrix or sometimes it only fills the partial crack as the healing agent is limited in the capsules. To save the material from failure and to preserve the mechanical properties, multiple healing was developed with the excess healing agent in the form of hollow fibers and microvascular known as mesoporous network employed in the matrix [44]. The healing technique is similar to the microcapsules technique, as the healing agent is deployed in fibers and a network of capillaries. Hollow fibers can be incorporated in fiber-reinforced polymer composites as a substitute for conventional composite fibers. The main advantage of fibers over microcapsules is that fibers can be interlinked to form a chain network. This allows the healing agent to deliver into cracks more effectively and also these fibers can be interconnected in such a way that a large area can also be healed in a single flow only. However, the vascular network can be deployed in the matrix as a three-dimensional system (3D), which provides the advantage of healing the crack in multiple directions in a single application only. Primarily there are two types of approaches for fabricating vascular composite materials, one of the common ways is to interconnect the hollow fibers from the vascular network and another approach is implementing a mesoporous network [45]. Out of all extrinsic techniques, the vascular network has the advantage of multiple healing cycles and a quick flow of healing agents into the crack. A lot of research is carried out in developing vascular networks as they are difficult to process, design and complexity in fiber reinforcement in matrix.

### 1.2. Reversible Cross-Links

To obtain excellent mechanical characteristics such as high modulus, solvent opposition, and high break energy, cross-linking, which is not a reversible procedure of polymeric materials, needs to be performed. Excessively cross-linked materials are brittle and appear to shatter and have an effect that harms polymer refabrication capacities. Reversible cross-links are used in polymeric systems [46]. Recently, the authors presented a method for creating cross-linked processable polymers. Reversible cross-connections, in addition to refabrication and recyclability, enable self-healing capabilities. The crosslink that is a reversible device, nonetheless, will not reveal its intrinsic self-mending abilities. An outside stimulus such as heat, light, or chemical triggering is necessary to get reversible action and subsequently, self-healing capability. Hence, these elements explain the notion of automatic non-recovery [47,48,49,50,51,52,53].

#### 1.2.1. Diels–Alder (DA) Reactions

Several polymers are being formed by using DA reactions, through reversible thermal polymerization. Cross-linking polymers being furanic maleimide or polymers with low-temperature maleimide pendants, come under this category. The retro-DA reaction happens at high temperatures, debonding the chemical associations of formed companies and reversing the cross-linking mechanism. The highly explored thermally driven covalent bond formation is DA reactions (4 + 2 cycloadditions), thus, a variety of studies on reversibility are available and were initially applied to this technique to fabricate thermally reconfigured polymers [54,55].

**Figure 5:**
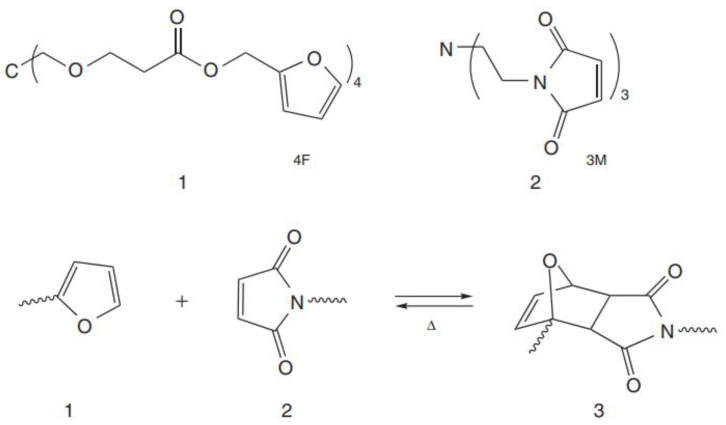
Schematic representation of highly cross-linked polymer [3M4F] formed by DA reaction using monomer 1 as multi-diene (4furan moieties 4F) and monomer 2 as multi-dienophile (3M) [55].

**Figure 6:**
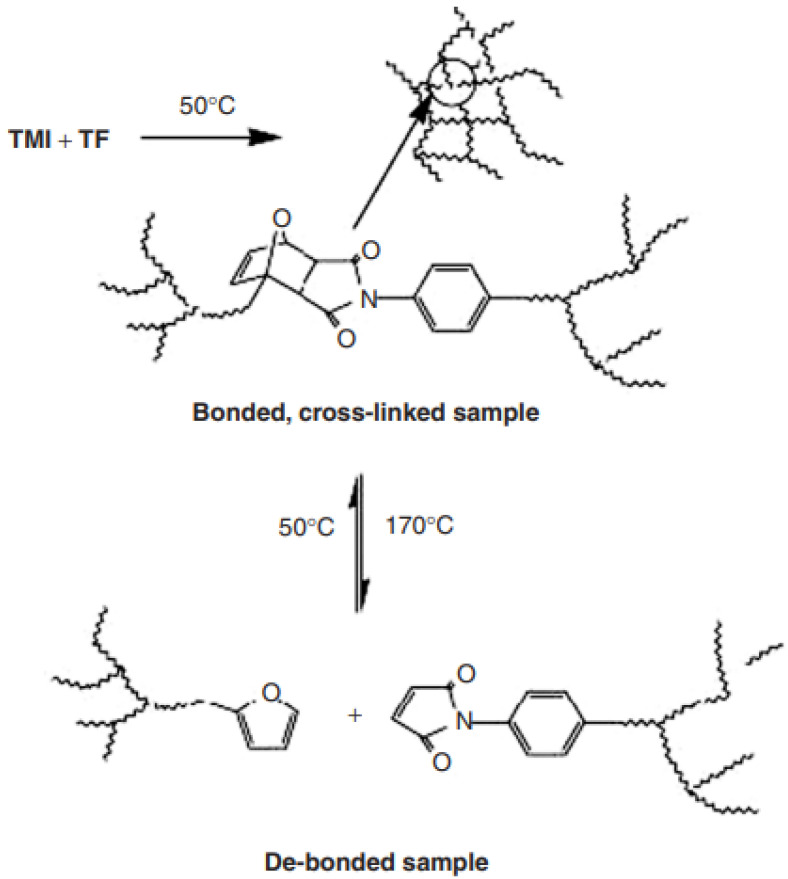
Reversible cross-link thermal reaction between TMI (trimaleimide) and TF (trifuran) [71].

The prior polymer synthesized (3M + 4F = polymer (3M4F)) (Figure 5), reveals a recovery of 53 percent strength [56]. Later, 83% of strength was recovered. The improved strength helped regain the mechanical system. Various other academic teams over the world have given more to this promising area of research since their discovery [57,58,59,60,61]. Bekas et al. [62] synthesized maleimide-containing polyamides-based polymers and a compound of tri-function furan. The adduct produced by combining the DA and retro-DA methods had high thermos reversibility and gel formation (Figure 5 and Figure 6). They recently exploited DNA and retro-DA mechanisms to produce self-healing capabilities using modified polyamides with varying concentrations of maleimide and furan pendant groups [63]. However, due to low mobile cracks, the designed product did not demonstrate complete healing.

Numerous researchers [64,65,66,67,68,69,70] recently reported light-induced crack healing properties. To obtain self-healing properties, they selected 2 + 2 photochemical cycloaddition of cinnamoyl groups. A photo cross-linkable 1, 1, 1-tris (cinnamoyloxymethyl) ethane (TCE) cinnamate monomer was used for their analysis. The mechanisms of cross-linking through cinnamoyl groups (photocycloaddition and recycloaddition) are seen schematically in Figure 6.

The process was validated using Fourier transform infrared (FTIR) spectroscopy, and the authors analyzed the complex self-healing capabilities by comparing the flexural power of the broken and restored samples. Photochemical healing is swift and does not need the use of catalysts, chemicals, or heat.

#### 1.2.2. Ionomers

Ionomers are polymeric particles with a hydrocarbon backbone and a pendant acid that may be neutralized to form salts [72]. Ion concentrations in ionomeric polymers or ionomers vary widely; although they typically range from 5% to 15% mol. Techniques for ionomer synthesis might be partitioned into two groups: direct synthesis and post-functionalization of the preformed polymer that is saturated

Ionomers have a large number of electrostatic connections between anions such as carboxylates and sulfonates and group 1A, 2A, and even electrical conductive metal material cations.

**Figure 7:**
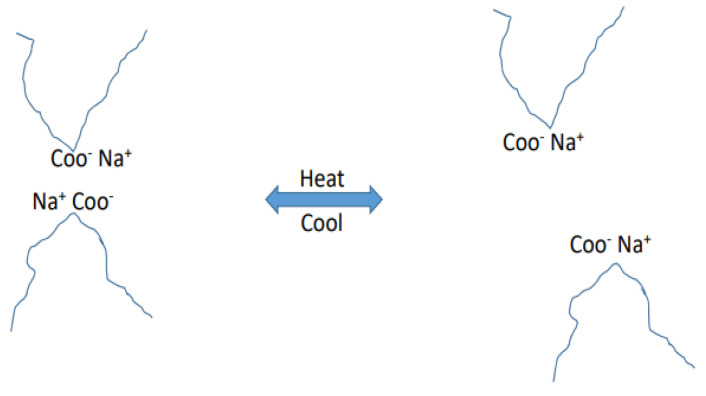
Reversible iconic interactions [6].

Transitional metal cations will find a broad range of sulfonates, carboxylates, and ionomers, including the duo of sulfonated and carboxylated groups in the same chain. Despite the opposite inclination of chain elastic forces, the ionic polar groups produce the result based on electrostatic interventions. There are ionic groups with reversible physical cross-links (Figure 7). The application of a limited number of ionic groups resulted in dramatic changes in the properties of polymers, such as tensile, shear, energy absorption, and abrasion resistance. They can be processed in the same way as thermoplastics, since ionomers are not thermosetting materials. This unusual merging of physical properties and simple processing has contributed to the use of this category of polymers in food and membrane packaging, separation, roofing bricks, parts for vehicles, covers for golf balls, coatings, etc. The reversible aspect of iconic bonds makes them ideal for engineering self-healing polymeric structures in addition to the above-mentioned applications [73,74].

#### 1.2.3. Supramolecular Polymers (SP)

In typical polymers, properties are obtained because of the long monomer chains that are bound together by covalent bonds. Monomers with lower mass were recently clubbed via reversible non-covalent combinations to produce polymer-like rheological and mechanical characteristics [75,76]. It is broken reversibly using noncovalent interactions and can be a special class of macromolecular compounds, known as supramolecular polymers. They display additional characteristics under thermodynamic equilibrium relative to regular polymers.

**Figure 8:**
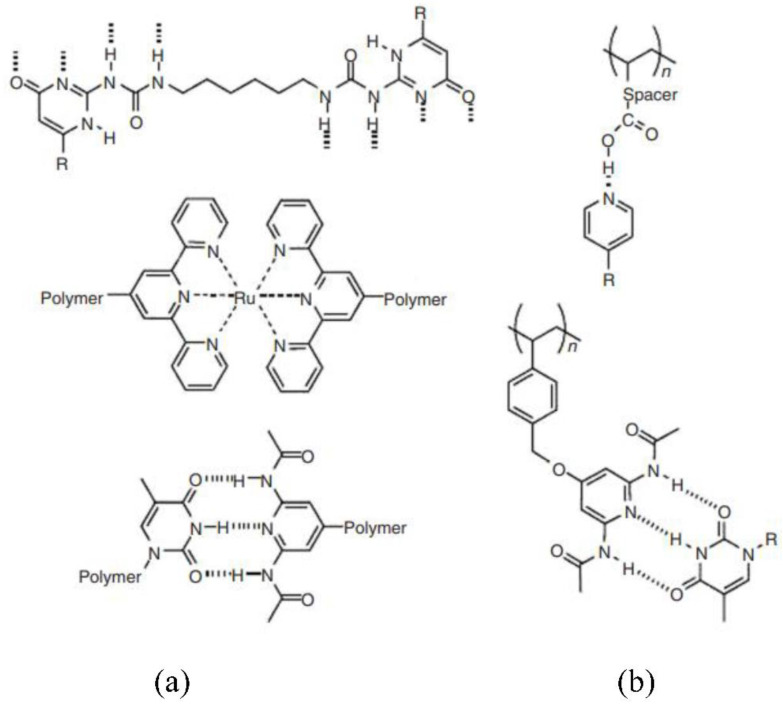
(**a**) Main chain SP (**b**) side chain SP [86].

Among these characteristics include environment-dependent switchable attributes, enhanced processing, and self-healing activity. Main chain and side chain forms are the two types of supramolecular polymers. Noncovalent interactions are used to improve or functionalize main-stream covalent polymers in the case of side-chain supramolecular polymers, since they protect the backbone of main-chain polymers, which are supramolecular. Figure 8 illustrates many renderings of both kinds of supramolecular polymers [77,78,79,80].

Different assembly forces are employed to generate supramolecular polymers, such as metal-ligand inter-combinations, π-π (pi-pi) interactions, hydrophobic, electrostatic inter-combinations, and chemical bonding is a kind of hydrogen bonding. The most common method for creating supramolecular polymers is hydrogen bonding. Finding the correct balance between a continuous connection and a reversible mechanism is the key problem with this technology. The bigger the association constant, the smaller the reversible connection. Conversely, the greater the reversibility, the lower the real constant with smaller assemblies and poor mechanical characteristics [81,82,83,84,85].

Major classes of thermally reversible polymers were made of Diels–Alder reactions, where in low-temperature applications cross-linking of furanic polymers with maleimide or polymers with maleimide pendants were considered. However, for high-temperature applications, retro-DA reactions evolved by debonding the chemical linkages of existing networks and start to yield in reverse by cross-linking. The 3M4F polymer is the first polymer designed for thermally re-mendable application and observed a strength recovery of 80–90% [70,71,72]. However, in the case of ionomers, hydrocarbon acts as the backbone and the existence of pendent acid tends to form in salt due to partial or full neutralization. As ionomers tend to act like thermoplastics and the presence of iconic groups tends to produce reversible cross-link interactions and aids in healing mechanisms which can be applied in the food packaging industry, roofing, automobile parts, etc., [76,77,78]. Whereas supramolecular self-healing composites have a sticker-like function that can reconnect via reversible behavior. Upon the mechanical damage, the weaker bonds break first and combine with newly generated interfaces containing sticky groups which can heal the material. The main disadvantage of supramolecular polymers is that they are not suitable for high-end structural FRP composites and aerospace applications because of their poor mechanical performance and low glass transition temperatures [85].

### 1.3. Miscellaneous Technologies

In the literature, technological advancements in self-healing methods are available. The following sections of the analysis address these new developments.

#### 1.3.1. Electrohydrodynamics

The mechanism of blood clotting was replicated by Ahangaran et al. [13] using colloidal particle aggregation at the faulty spot. Employing the electrohydrodynamics (EHD) flow theory created self-healing materials. They used a colloidal particle suspension confined within the two walls of a metal cylinder. During the experiment, a conductive coating is applied to the walls, followed by a ceramic insulating layer. The electric field is applied to this device via a concentric metal wire. The current density at the affected spot grows when the separating sheet is damaged, allowing colloidal particles to congregate at the issue spot due to EHD flow.

Since the gaps between colloidal particles impede the creation of a thick crust, the accumulation of particles is inadequate to fix the flaws. The author recommends adopting polymeric colloidal particles or an anode that is simultaneous sacrificial electrodeposition of this metal at the issue zone to improve data recovery performance [87].

Self-healing is achieved by components such as thermo-reversible polymers, dietary fiber insulation, and electromagnetic cables. The interlaced cables function as electrical and thermal conductors, evenly distributing temperature. Additionally, the excess fibers added in the process help in the healing process. For instance, when non-positive CTE fibers are used to fill the center of this braid or the weave that is laminate, it will shrink when heated. The damaged polymer (which has a good CTE) expands whilst the reinforcing fibers compress, leading the matrix to condense around the crack [88,89,90].

#### 1.3.2. Conductivity

Polymeric materials are intrinsically isolative. These polymeric materials can be improved for electronic applications by imparting conductivity to the systems. Tunable conductivities in polymers may provide structural integrity through electrical feedback, which could assist in the arduous procedure of discovering and distinguishing microcracks. As a result, for materials with conductivity, the aptitude to self-heal could be beneficial in deep space or ocean applications. In polymeric materials, on the other hand, conductivity could be exploited to produce capabilities that can be self-healing, regarding their electrical conductive characteristics. Fathy et al. [91] utilized organometallic polymers according to N-heterocyclic carbenes and transition metals. These polymers show outstanding processability and structurally robust properties within the solid state. Reversible systems have an electrical conductivity of 10^−3^ S cm^−1^, and their share in the construction of conductive self-healing materials could be shown schematically. The amount of electron percolation paths decreases, and electron opposition increases each time a microcrack does occur in a structure. Whenever depolymerization occurs, the introduction of bulky moieties of n-alkyl carbenes can reduce the viscosity steadily; therefore, the flow to the cracks increases. In addition, to provide practical applications that can be self-healing, higher conductivity (1 S cm^−1^) should be acquired. Multi-walled nanotubes (MWNTs) have been successfully incorporated in cup composites with fiber-epoxy [92]. A minimal concentration of carbon nanotubes (0.1 percent by fat) is found to be adequate to generally meet the percolation threshold within the composites. The composite matrix’s epoxy MWNT can reliably identify the onset, existence, and development of injury. This characteristic can be helpful for extensive purposes, including the evaluation of approaches for self-healing.

#### 1.3.3. Shape Memory Effect

The most used shape in which plasticity deformation is applied to the minor temperature martensite phase is virtually totally reversed while transitioning towards the temperature austenite period and it is proven by several highly purchased intermetallic systems [93]. These systems form self-healing memory (SMAs), which could be exploited in several applications. When heated, SMAs such as, for instance, Nitinol (nickel-titanium) indicate a self-healing process that is possible [94]. They might return to their original state if deformed and heated at maximum conditions.

#### 1.3.4. Migration of Nanoparticles

Balazs and colleagues (2006), have explored that cracks are sealed by segregating nanoparticles in fluid because of the polymer-induced depletion involving the surface and particles [95]. The morphological analysis gained from the simulations of molecular dynamics was utilized to assess the efficiency of self-healing a lattice spring replica. The model received predicts mechanical property efficiency in a lattice spring replica. It predicts mechanical property restoration of up to 75–100%. Self-healing materials have yet to be demonstrated based on the latter methodology—the introduction of polymeric structures of nanoparticles.

It has two advantages: it increases the system’s mechanical power and segregates the crack’s surface. Due to its superior mechanical approach, CNT is one of the fine materials to use for self-healing purposes based on this method, compared with other particles.

#### 1.3.5. Co-Deposition

**Figure 9:**
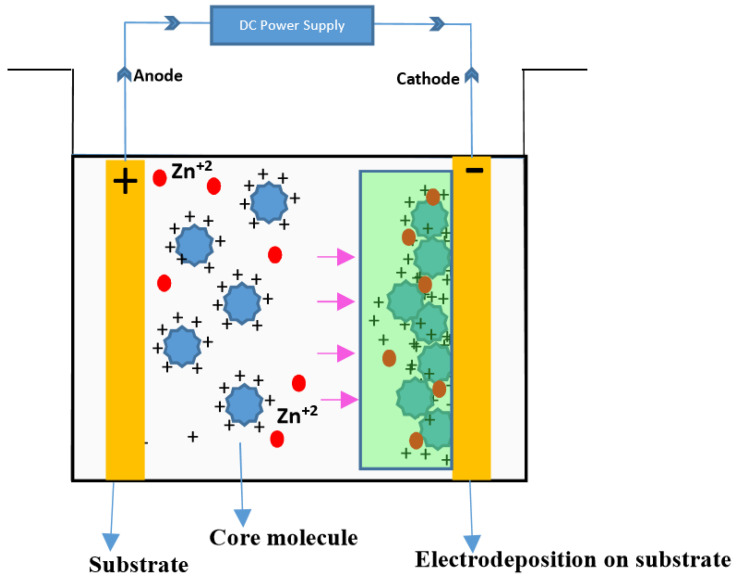
Electrolytic co-deposition of microcapsules [99].

To come up with self-healing anticorrosive coatings, electrolytic co-deposition can also be employed. With this system, microcapsules corrosion inhibitors may be integrated into composite plating. Being a product that is initiating micro- or nanocapsule production, fluid corrosion inhibitors or mesoporous nano-materials are inbuilt with absorbed corrosion inhibitors that could be exploited [96,97,98]. These capsules will be coated with metal ions such as Zn^+2^ and Cu^+2^ to produce composite metallic layering, as shown in Figure 9.

In addition to the self-healing methods listed above, various novel ways to manufacture self-healing materials are projected to emerge in the near foreseeable future.

### 1.4. Applications

Industry product marketing is generally classified according to the following points:

1. “Concept development (preliminary level)
2. Laboratory execution (product level)
3. Pilot line upscaling (process level)
4. Industrial applications (marketing level)” [100]

Self-healing materials are within the very early phases of research (preliminary level or product level). Consequently, they are not yet marketable; however, self-healing materials are expected to be employed in nearly every area. Only a few applications have been created, primarily in the automotive, aviation, and construction industries. Nissan has developed the world’s very first transparent automobile finish that is self-healing.

To trade this product, they named it “Scratch Guard Coat”. The company also stated that this hydrophobic paint repairs the scratches on coated surfaces that occurred during a car wash, off-road travel, or occurred by hard objects for a tenure of 3 years. This newly developed high-elastic polymer paint prevents scratches from reaching a car’s internal layers. This complete recovery takes from one to seven days, considering the size, scrape, and temperature associated with the adjacent area. The two polyurethane materials that are used as component coatings from Bayer Material Science provide another instance of this type.

In thermoset polymers (proportional to plasticity and elasticity relation), the energy needed to control the resistance of the material to generate an abrasion is larger than in thermoplastic polymers (viscoelastic reaction). When a scratch appears on a material, it causes the substance to migrate from the area that is damaged, the groove’s side. Generally, in thermoplastic polymers, energy loss happens in viscous flow in the absence of residual stresses, and surface tension helps to reflow the material to the side of the groove. In thermosetting polymers, the energy used to form a scratch is maintained in the conduit industry (below the strength for the yield). The accumulated energy is released once the mechanical force is released. Additionally, the twisted polymer chains return to the groove. This repairing technique is critical to the movement associated with the polymer chains used in their glass transition temperature.

The aircraft sector is projected to be the next sector which is the industrial usage of self-healing materials. In the last few years, composites have become increasingly predominant in airplanes. Hollow fiber-reinforced composites may be a remedy that is feasible for restoring fractures and accidents. Self-healing polymers have made room for practical uses [101].

The building industry provides prospects for various self-healing materials. To start with, real self-healing is just a plausible solution that may be completed rapidly. Self-healing inhibited along with corrosion-resistant coatings for metallic structures, such as steel might help reach long life service while minimizing maintenance expenses. Medicinal sections describe some areas where self-healing drugs can be utilized. Synthetic bones, teeth, and other prostheses can last longer thanks to biocompatible self-healing that is composite. Self-healing plastic, an innovation that is reasonably brand new, might find use within the toy industry. Finally, it is feasible to claim that the technology necessary to make self-healing items are costly and therefore limits their applications in industries. Shortly, emerging technology will enhance the utilization of self-healing materials in our everyday lives.

## 2. Self-Healing of Metals and Metal Matrix Composites

It is a difficult assignment to build self-healing metallic materials. Repair procedures tend to occur at higher temperatures or in extreme conditions because of the high melting temperatures of metals which easily form oxides on surfaces through bonding with oxygen molecules on and off water surfaces. There are, however, some methods currently being developed to include metallic and metal matrix composite structures with self-healing properties. This comprises strong state techniques, namely rainwater healing, semisolid phase ideas, such as electroplating, and volatile alloys having a low melting point, similar to liquid state ideas, such as electroplating and vascular networks filled. Mates are increasingly important in fabricating components with self-repairing characteristics taken from nature in inorganic structures. While significant progress has been achieved in self-healing polymers [102,103,104] and ceramics, progress in self-healing metals has been slow. Methods to fabricate metallic structures are not to be directly copied from natural techniques, as this may lead to many problems. These methods for metallic structures should not be directly mirrored by the natural techniques that motivate them, posing many problems. Living systems create healing substances, which are subsequently delivered to the injured site and utilized for restoration. A high temperature and an extended amount of time are also essential for solid-state processes to heal. Metallic bonding is impeded by oxidation instead of ceramic or polymer-based materials since fresh metal oxidizes when exposed to the atmosphere or liquids, making bonding much more difficult. Since these repairs need high temperatures, the melting point must be performed at even greater temperatures.

### 2.1. Classification of Metallic Structures in Self-Healing

**Figure 10:**
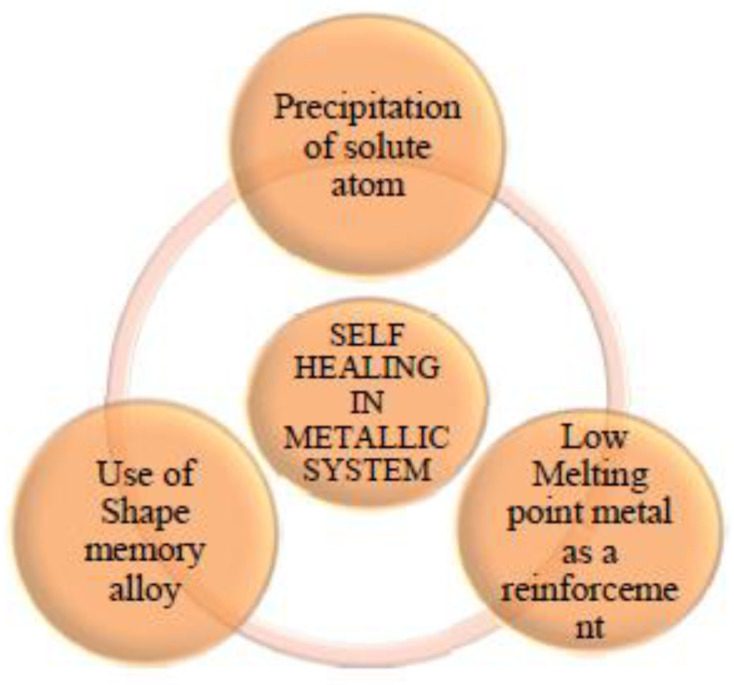
Methods of self-healing metals [113].

The self-healing procedures of revolutionary metallic materials are classified according to their product composition or autonomy (Figure 10). Additional actuation, such as applying heat or electricity, is essential for non-autonomous self-healing systems. Even though autonomous self-healing materials do not require outside actuation, autonomous metallic self-healing systems continue to be mostly theoretical currently, due to a lack of noticeable experimental results. Self-healing metal matrix composites (MMCs) are homogeneous macroscopic materials, formed from memory alloy (SMA) fibers and encapsulated healing chemicals commonly spread in a matrix. Irrespective of size, SMA wires are already placed in metal matrices (Al, Zn, Sn, or alloy that is Sn-Bi) as reinforcements to produce self-healing materials centered on SMA. Self-healing, depending on encapsulated recovery agents, is established by injecting capsules or pipes carrying a representation that is curing as solder [105,106,107,108]. Recovery through coatings, electro-heating, eutectic-based, and precipitation-based recovery are typical examples of self-healing [109,110,111,112]. Theoretical research focuses on nano-scale processes, including nano-SMA and ground boundary migration self-healing metals.

### 2.2. Mechanisms of Self-Healing Metal and MMCs

#### 2.2.1. Precipitation-Dependent Healing

In precipitation-based recovery, micro-cracks/voids in the substance behave as nucleation areas for supersaturated or under-aged alloy precipitation. Solute atoms happen to be defects and voids in underaged alloys, in essence, “healing” them. This procedure shows the void as the outcome of being filled by migrant atoms in self-recovery and being examined. This healing, nonetheless, happens on a nanometer scale level comparable to the standard age-hardening process; however, it cannot fix heavy cracks. Precipitation occurs at microcracks that generate precipitates within the localized, highly stressed location, and recovery can be expedited by warming an alloy to an aging temperature that is particular. [114]. If the alloy cools, it becomes supersaturated meta-stable from high heat, and then returns to equilibrium, which happens during aging by precipitating supersaturated solutes over voids and cracks. An under-aged alloy such as Al-Cu-Ag-Mg increases its creep resistance by heat treating at high temperature but at a constraint rather than fully hardened, e.g., to T6 temper, dynamic precipitation of the following alloy even at 500 h creep at 300 MPa and 150 °C, even associated with dislocations and healing of the cracks, is obtained by further aging of heat treatment.

The curing of the break triggered additional heat that is aged. Precipitation heat treatment impacts the crack action in Al-Cu AA 2001 alloy in under-aged boundary situations, which has been investigated [115]. It indicates the microstructures of the under-aged alloy were crack healed after the aging treatment for 10 h. The work [116] studied the precipitation of boron nitride (BN), which leads to a self-healing mechanism in 304 stainless steel improvised with boron (B), cerium (Ce), and titanium (Ti). The alteration of B, Ce, and Ti of the alloy contributed towards the preferential BN accumulation in the locations associated with the creep cavity, and it is stable at high temperatures at creep cavity surfaces and leads to an increase in the creep resistance of 304 stainless steel. This procedure, as conducted over voids, holes, and other free surfaces, is heterogeneous to attain self-healing, which will behave as nucleation in odd cracks. Researchers examined how precipitation heals in two different areas. Precipitation recovery at high temperatures (575–750 °C) was mostly examined on stainless steel and Cr-Mo-V alloys, although it was mostly studied on Al alloys at low conditions (120–185 °C). Diffusion, which is the rate-constrained site that can manage the motion of atoms to the matrix and into gaps and fissures in the matrix, requires some time to accelerate. Precipitation-based recovery has thus far been limited to small-scale injury by studies. This can remove fatigue fracture start sites over extended periods, although restricted to early harm stages. Precipitation-based mending could have a small effect if a break widens.

#### 2.2.2. Healing Based on Nano-SMA-Dispersoids

Nanosize shape memory alloy (nano-SMA) dispersoids were suggested for self-healing metallic materials. Period change of SMA nanoparticles, which belongs to the grouped community of nanoscale recovery processes, fills nano-voids. Few authors stated that the essential idea is currently in its very early phases of development. Additionally, the capability to self-heal has yet to be proved. The microstructure is first made up of a host matrix with embedded coherent SMA nanoparticles stabilized by the host matrix in its austenite phase which is a high-temperature zone [117,118,119,120,121]. Whenever damage is generated by dislocation localization and nanovoids are formed, activation of nanoparticles occurs, and the nanovoid strain is expected to change the phase transition of the SMA nanoparticle from austenite to martensite [122]. The change of phases is followed closely by a sharp change in the shape of the particle that produces regional stress areas on the host matrix and plays a part in the closing of cracks.

Theoretically, when small-size nano-dispersions fill voids, it can cause residual stresses and alter the fatigue characteristics of the material. The unit’s absence of bonding capabilities could be a performance impediment on the other side.

#### 2.2.3. SMA-Based Healing

Self-healing materials with memory components aid in regenerating bulk geometry after having a fracture which is a feature not found in other self-healing modes. This is crucial regarding restoring a building’s previous functionality after significant damage. SMA reinforcements are utilized in selected steel matrices for self-healing in this technique. Shape pseudoelasticity and memory are two characteristics that distinguish SMAs. Whenever heated above its austenite transformation temperature, an SMA’s ability to deform into the martensitic state and then return to its original shape is known as pseudoelasticity [123]. The latter is connected with an SMA that can recover the strain which is highly applied upon unloading the material when it is in its austenite phase. In self-healing materials, heating at a high temperature helps to achieve substantial recovery by constraining shape recovery, which can lead to geometric reconstruction and crack closure. SMA has been enhanced to aid the healing process; nevertheless, the services and products remain essentially non-autonomous and need external actuation (typically heating).

The primary challenges encountered by self-healing SMA-reinforced materials, are (I) maintaining the bond between the SMA and matrix. (II) Synthesis affinity between SMA and the steel matrix; (III) The characteristics and recovery kinetics of SMA-reinforced matrix. (IV) SMA-metal matrix compatibility during synthesis; (V) An intensive knowledge of the dynamics and recovery kinetics of SMA-reinforced matrix; (VI) A clear understanding of the damaged area and how to speed the recovery process up. A self-healing material that is tin (Sn)-based was created using a strengthened Sn-21 Bi (wt. %) matrix alloy with 1% equiatomic NiTi SMA wires [124]. The composite, which contained a matrix that is complete in a tensile test, cooled to atmospheric temperature, and tested again, was fixed within an oven at 169 °C for 24 hours. The break, along with the restored specimens, was indeed found to be closed. It was unearthed that 95% of the original tensile strength was recoverable. The design was preserved by warming the SMA and softening the matrix solders. During heating, NiTi cables started to stress the matrix to recover to their original form (shorter lengths) The usage of other alloys as matrix materials for self-healing components has additionally been investigated. In experiments on healing, in a matrix that is magnesium-dependent partial recovery can happen at a given temperature. Qiao et al. [50] investigated the utilization of NiTi SMA wires within an Al-A380 matrix. Due to a lack of adhesion between the cable and the matrix, SMA cables alone were not able to repair the damaged faces. Wrapping SMA wire along the threaded stainless steel and casting Al-A380 around the rod/wire addressed the challenge. This rod acted as a technical anchor, allowing the SMA to pull, although the whole cable had disintegrated. It was discovered that the strengthened component was approximately twice as strong and ductile due to the unreinforced sample. Since the re-bonding was inadequate, there was no significant power, although a significant decrease in fracture width and residual compressive stresses was observed. Because SMA is now a part of self-healing, limited rehabilitation may now be used to produce recurring compressive stress fracture repair. This residual load by post-tensioned tangible systems enables the structure to withstand externally induced stress without bonding. Continuous initiatives are fond of describing this possibility in terms of how healing systems may be constructed to withstand axial or external bending stresses. Since applied stress rises, adhesive bond energy grows; this capacity is thought to aid bonding in self-healing.

#### 2.2.4. Encapsulated Healing

In this section, the healing performance of capsule-based self-healing materials is discussed. Table 2 shows the healing performance of capsule-based self-healing materials. Microcapsules containing dicyclopentadiene (DCDP), a monomer curing representative, were embedded inside a polymeric epoxy matrix, including a catalyst to achieve the self-healing of autonomous polymers. Self-healing happens when capsules that discharge the agent (a monomer) are propagated and broken by a break in polymeric materials. Encapsulated agents treatments, such as polymer healing agents, have been utilized to create self-healing MMCs. Ferguson et al. [125] proposed the growth of the latest MMCs being self-healing encapsulated solders in 2008. Materials with low melting temperatures were encased in ceramic shells to identify the breach and then scattered within a host matrix with a greater melting temperature [126,127,128]. After heating, the solders had low temperatures and flooded into the fracture, charging the space by capillary action and starting bonding at particular conditions. The energy data recovery with this curing procedure depends on this host steel structure; therefore, the properties of alloys having a low melting temperature exhibited an energy restoration of 60% associated with the initial pre-damaged energy [129,130,131,132].

#### 2.2.5. Coating-Based Healing

The authors created the facet of the titanium alloy with a thickness of 2.03 mm, a 60 percent indium–40 percent tin (wt. percent) self-healing coating with a melting temperature of 124 °C and a thickness range of 0.005–0.0015 mm. If a surface break appears, these devices may be heated through the In–Sn alloy’s melting point. As soon as the specimen is heated, the break in the titanium alloy is covered by molten area alloys [133,134,135,136]. Crack healing evaluation revealed that after the procedure, which is a self-healing process, crack development is avoided by employing a low crack-tip driving force. An increased crack-tip driving force can result in a 50% reduction in the break development rate. The self-healing coating could be activated repeatedly, indicating the chance of multicycle heating in inert conditions.

#### 2.2.6. Electro Healing

Steel ions are electrodeposited onto a fracture in a bath with electrolytic-regulated electric currents in pure nickel sheets, resulting in break recovery. The authors examined electro-healing. The cracks with sizes up to 100 µm within the micrometer range had been effectively healed utilizing the procedure of electro-healing. This action has regained almost 96 percent of tensile power. While this option would be effective, there could be a limitation to the need to place a framework combined with a small scale of heating.

#### 2.2.7. Eutectic-Based Recovery

**Figure 11:**
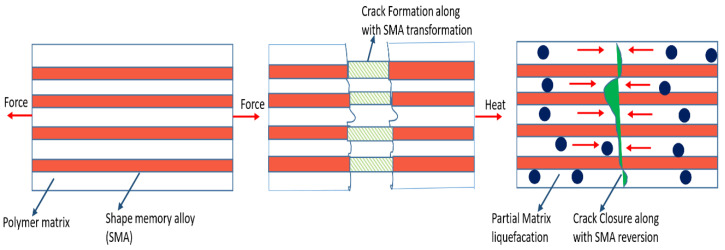
Liquid-aided healing of MMCs reinforced with SMA [141].

Numerous researchers [137,138,139,140] examined eutectic-based healing using eutectic liquid formed in its solid phase and acting as a healing region, while the solid dendrites are composed of structural integrity. The best path to accomplish this is to use a matrix that is distant from eutectic composition to form eutectic dendrites at higher temperatures. When the melt is cooled, dendrites of this main period are created because of their high melting temperature. These dendrites deform the structure of the remaining inter-dendritic fluid, repelling the dissolved substances, so the composition of the inter-dendritic fluid changes. To achieve this for self-healing, the temperature has to be increased until the interdendritic eutectic melts and flows into the specimen through the mechanism of cracks and then closes the crack, while the hard dendrites keep the structural coherence of the system. The fluid is a eutectic movement involving the dendrites and enters any holes or gaps within the unit; the eutectic liquid solidifies in the cracks and heals the specimen, as shown in Figure 11.

### 2.3. Applications and Future Scope

The attractive advantages of the usage of self-recovery in metal substances include low renovation costs, lengthy carrier life, and avoidance of catastrophic remarks. However, self-recovery in metal substances has not been extensively utilized in real-international applications, particularly for load-bearing applications. MMCs with self-recovery traits are being evaluated properly now. Self-recovery metals and substances are not blanketed in any purchaser products. Self-recovery polymers crafted from MMCs, which include self-recovery slicing pads, are currently commercially viable. Nonetheless, self-recovery metals and self-recovery MMCs have several uses. In the renewable strength and biomedical sectors, self-recovery metallic substances with sturdy mechanical properties can be created for wing parts, structural components, spinning components, blades of turbines, and steel implants.

## 3. Self-Healing Ceramic Matrix Composites

### 3.1. Introduction

The manufacturing equipment of ceramic matrix composites can cause fractures. The fiber architecture, the effectiveness of the fiber/matrix connection, and the stress collectively affect the density and apertures of those microcracks [142]. The smaller the fracture density, the lower the shear stress at the interface of a bigger fiber/matrix aperture [143,144,145,146]. These cracks in the matrix provide a path for oxygen to scatter to the fibers and interphase (micro-cracks will also be considered). The interphase is a slim finish, which includes carbon graphite, situated on the fibers to redirect matrix fractures through the fibers without splitting them, transforming the cracks from I to II mode. In addition, if the fibers turned extensively oxidized and responsive to sub-critical fracture, their thermo-mechanical opposition may drop significantly as time passes. When fibers stick along with the matrix during oxidation, then oxide fills the gap between the fiber and matrix, which results in degradation. These trends are proportionately due to the oxygen fuel line that diffuses through the bulk of the composite material. Self-recovery should be integrated into composites to extend the lifespan of thousands of hours at elevated temperatures in corrosive and oxidizing conditions, as applied in contemporary jet engines. The durability and protection of diverse structures can be enhanced through the self-recovery approach [147,148,149,150,151].

### 3.2. Analyzing Self-Healing

Ideally, a self-healing device will have the capacity to restore damage multiple times so that it can be automatically fixed as new cracks emerge. Self-healing is accomplished by preventing or restricting oxygen entry into the substance. This allows for the filling of a crack by a condensed phase by absorbing a portion of the entering oxygen. Self-healing can occur on the inside or outside of ceramic matrix composites (CMCs).

#### Requirements of Self-Healing CMCs

Using a multi-component functional matrix that is ceramic is a promising technique for creating non-oxide ceramics that can be both durable and oxidation-resistant, with the capacity of shutting matrix fissures. The chemical vapor infiltration approach will accurately deposit each constituent as a homogeneous finish causing a multi-layered matrix to manufacture these CMSs. You can also follow a slurry cast’s path. All materials must withstand serious conditions, although temperature ranges must be diverse. The sealing glass phase formed in situ should have a decreased oxygen penetration to prevent air entry to the fibers.

### 3.3. Modeling of the Matrix Interphase Architectures

#### 3.3.1. Existing Interphase Materials

When constructing the fiber/matrix binding zone or inter-zone, all of the application’s criteria should be considered, including high temperature and oxidizing conditions, as well as a range of thermo-chemical and thermo-mechanical compatibility concerns. Even though the nature of a self-healing matrix usually covers the fiber/matrix interface, pyrocarbon should not be employed as an interphase-mediated material because it will react strongly with oxygen. An oxide layer, such as layered perovskites, monazite, micas, or βalumina/magnetoplumbite structural materials is suited because the mixed zone cannot further oxidize.

Any micro-cracking, twinning, or dislocation glide cleavage combination will result in considerable elasticity due to its crack deflection potential. MAX phases are ideal for interphase materials because of their layering figure, high refractory, great oxidation control, and self-healing capacity [152,153,154].

A typical treatment for enhancing oxidation resistance and interphase self-healing is to add boron to pyrocarbon. Boron addition has two advantages: it boosts the pyrocarbon interphase’s oxidation resistance, with the highest advantage being less than 8% for the boron atomic ratio, and (ii) it improves pyrocarbon anisotropy. Another possibility is to use a boron nitride interphase instead of the pyrocarbon interstate. The thermomechanical characteristics and lifetimes have improved since the BN interphase shields every fiber separately. (iii) At the BN/SiC matrix intersection, matrix fractures are deflected. In a tow, load transmission to fibers through the matrix is homogeneous, and oxygen diffusion via a matrix break only at the fracture region, preventing further material degradation [155,156].

#### 3.3.2. Multi-Layered Interphase

**Figure 12:**
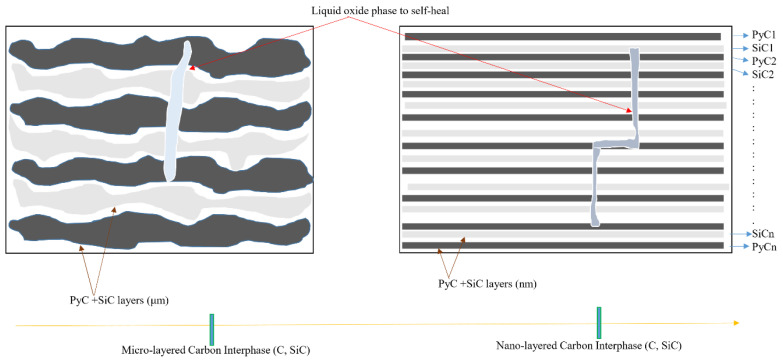
Interphase thickness between nano-carbon layers [157].

Variouslayered interphases were created by alternating a crack-deviating substance (such as pyrocarbon or hex-BN) with a component that brings upon a condensed oxide phase when faced with an oxidizing environment such as TiC, SiC, or B4C, as shown in Figure 12. There are several advantages to using such interphases. To begin with, several fractures increase owing to the presence of various surfaces, which increases the chance of deflection. Second, oxidation-sensitive material is reduced on average; for example, SiC partially replaces pyrocarbon or hex-BN. When these interstates are faced with an oxidizing environment, the liquid oxide phase generated by BN or B4C oxidation flows with the fracture channels, causing them to self-heal.

#### 3.3.3. Self-Healing Matrix

**Figure 13:**
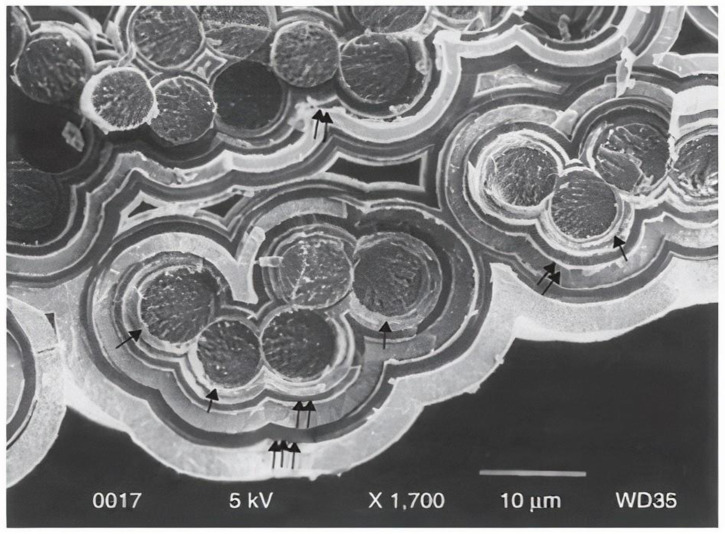
Self-healing with multi-layered SiC matrix deposited by pulsed CVI viewed in a transverse direction [162].

The boron-bearing stages within the SiC matrix are evenly distributed each time a slurry-casting strategy is implemented (initiating from the suspension of boron-based particles such as B_4_C, SiB_6_, or boron itself, either alone or blended with SiC particles) [158]. A CVI approach combines concentric levels of binary or ternary stages from the B-C, SiC, or Si-B-C systems (Figure 13) in a multi-layered SiC-based matrix.

At low temperatures (500–1000 °C), boron oxide stages are advantageous, but silica-rich stages are preferred (1000–1500 °C). When borosilicate fluid phases form in oxidizing environments, they quickly fill and spread matrix fissures in the material under strain. As oxygen diffuses through the fracture network, the oxide phases trap it, limiting diffusion to the interfacial zone of oxidation-prone fibers and the fiber/matrix interface, which increases the material’s durability [159]. Lifetime outcomes are highly important such as increases of at least one order of magnitude [160,161].

#### 3.3.4. Nano-Textured Matrices

Due to its potential to revolutionize material efficiency, nanotechnology is garnering much attention these days and microstructures now offer new options.

The slurry and sol (solution) processes are two of the many options for integrating nano-sized precursors. It eliminates the requirement for powder preparation and handling. It offers the liberty to mix simple composites and molecular variations, which makes this method highly desirable.

### 3.4. Analyzing the Characteristics of Self-Healing CMC

The function of hypothesized multi-layered self-healing matrix composite was to create a condensed covering and protective oxide layer when air stress in the environment is high enough. This isn’t always the entire instance, especially in circumstances with high moisture compared with oxygen amounts. Finally, the oxidation kinetic laws for every single product must be determined across a wide heat range to assess the self-healing capabilities of composites by using this multi-layer Si-B-C matrix that is ceramic [163,164,165,166].

#### 3.4.1. Corrosion and Oxidation Opposition Regarding the Constituent

Oxide shape needs to be calculated to comprehend the steady-state stagnation procedures and prices during paralinear oxidation and volatilization. Small alterations in response price, and, small alterations in pressure and gas velocity, have a significant influence on time constants. Hawaii’s steady rate of SiO_2_ formers in combustion settings in the paralytic regime is acceptably described by linear volatilization rate constants. In combustion situations, the steady-state oxide thickness, time constraints, and recession rate paths are employed to depict material character. According to the experiment, up to about 1100 ^o^C, Si(OH)_4_ appears to be the dominating vapor species. SiO(OH)_2_ species may be significant at greater temperatures (Equations (1) and (2) [167,168,169,170,171,172].

“SiO_2_(s) +H_2_O (g) ↔ SiO(OH)_2_ (g) (1)

SiO_2_(s) +2H_2_O (g) ↔ SiO(OH)_4_ (g)” (2)

Thermochemical stability of glass-phased protective boron oxide and borosilicate: Boron oxide possesses characteristics making it easy to fit a matrix micro crack, with low melting temperature (about 460 °C) and low viscosity. This oxide will respond with dampness to make an acid that is boric according to the following temperature calculations (HxByOz).

3/2 “H_2_O (g) + 3/2 B_2_O_3_ (l, g) ↔ (HBO_2_)_3_ (g) (3)

3/2 H_2_O (g) + 1/2 B_2_O_3_ (l, g) ↔ (H_3_BO_3_) (g) (4)

1/2 H_2_O (g) + 1/2 B_2_O_3_ (l, g) ↔ (HBO_2_) (g)” (5)

(HBO_2_)_3_ and H_3_BO_3_ are the main species at low temperatures (T < 900 °C), whereas HBO_2_ is the sole dominating species at elevated temperatures (T > 900 °C). The capacity of this oxide to self-heal is harmed as a result of the volatilization process.

#### 3.4.2. Resistance to Oxidation of SiC

Silicon-based ceramics and composites are developed for use in a range of combustion applications. The SiC matrix layers may be substantially oxidized in dry air at 1000 °C in high-temperature settings, resulting in a protective SiO_2_ scale:

SiC + 3/2 O_2_  → SiO_2_ + CO (g) (6)

SiC + 2O_2_ → SiO_2_ + CO_2_ (g) (7)

Moisture in an oxidizing environment escalates the built-in oxidation of silicon-based products compared to values reported in dry air or atmosphere [173,174].

This occurs because silica is much more dissolvable in liquid than oxygen. Oxidization and volatilization of SiO_2_ molecules are required to form the temperature dependence of self-healing of SiC particles as a function of gas velocity and pressure.

#### 3.4.3. Oxidation Resistance of Boron Carbide

According to reactions 3.8, 3.9, and 3.10, boron carbide is helpful for composite (of the Si-B-C-C) multi-component self-healing ceramic matrix system because it produces liquid oxide (B_2_O_3_) at temperatures over 500 °C. When the oxygen partial pressure falls below a certain level, reaction 16.6 kicks in.

“½ B_4_C + 2O_2_ (g) → B_2_O_3_ (1) + ½ CO_2_ (g) (8)

½ B_4_C + 7/4 O_2_ (g) → B_2_O_3_ (1) + ½ CO (g) (9)

½ B_4_C + 3/2 O_2_ (g) → B_2_O_3_ (1) + ½ C (s)” (10)

The volatilization of boron oxide is negligible in a dry atmosphere if the temperature remains below 1000 °C. For B_4_C, depending on temperature two oxidation mechanisms have to be considered. The oxidation of boron carbide at temperatures below 600 °C is followed by a complete evade of the carbon first contained in the deposit.

**Figure 14:**
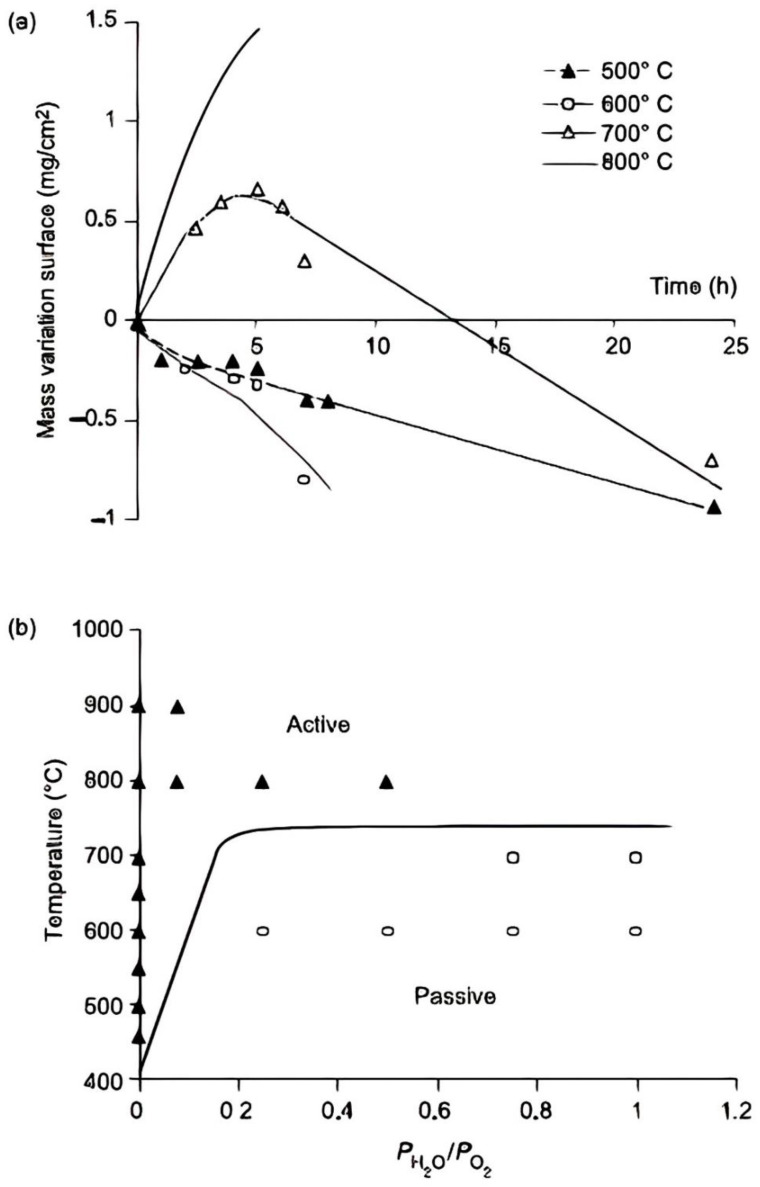
(**a**) Effect of temperature on B_4_C oxidation. (**b**) Changes due to the composition of the gaseous phase [158].

Nevertheless, some of that is possible at conditions more than or corresponding to 600 °C. During boron carbide oxidation, carbon stays in solids [175,176,177,178,179]. Significant volatilization of boron oxide happens in wet environments. Active oxidation of B_4_C can be detected at temperatures below 700 °C, dependent on both PH_2_O and gasoline velocity (Figure 14). Primary fat development is noticed, suggesting a passive regime at temperatures of about 700 °C. These data enable you to calculate the change heat for the composition and heat of the environment that is gaseous in the oxidation domains with a gasoline velocity of 0.2 cm/s. Utilizing the separate kinetic laws of B_2_O_3_ generation and volatilization, the experimental behavior can be easily repeated (using a paralinear model).

#### 3.4.4. Resistance to Oxidation for Carbide of Boron and Silicon

**Figure 15:**
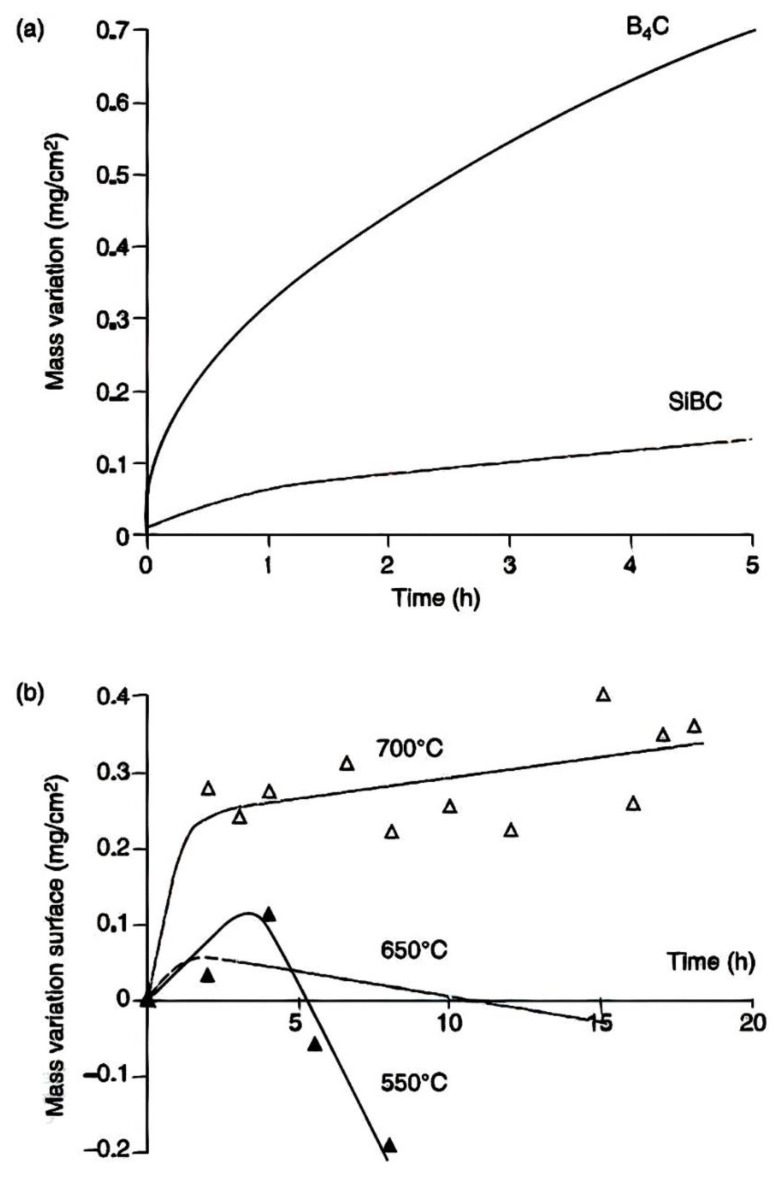
Change in weight during oxidation of SiBC in a furnace under the cold zone [158]. (**a**) Mass variation vs. Time and (**b**) Mass variation surface vs. Time.

As soon as the SiBC state is oxidized, a glassy borosilicate kind develops with high thermal viscosity and stability than B_2_O_3_ [180]. Due to the restriction of air penetration via this oxide scale, the SiBC process oxidation rate is a little slow (Figure 15).

The more silica content in borosilicate, the higher the oxidation temperature. Because of preferential volatilization, the SiO_2_ focus on the borosilicate oxide scale rises during the oxidation of SiBC in the presence of moisture. This enrichment assists in the development of an oxide scale that is resistant to both dampness and oxidation, where an upsurge in oxidation increases its protection. (Figure 15).

#### 3.4.5. Oxidation Testing of Self-Healing Matrix Composites

**Figure 16:**
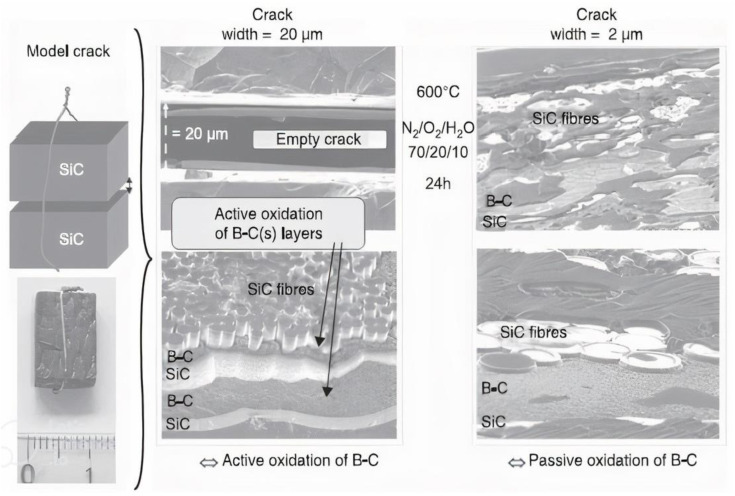
Self-healing in the model of planar crack at different ranges [162].

A planar model fracture has been machined on a small piece of CMCs to analyze the self-healing procedure in various moist environments (Figure 16). The composite was split perpendicular to 1 associated with the fabric’s major fiber directions, while the two sides in the machine were polished and then placed next to one other to create a single fracture by having a controlled kind. This led to a planar model break having a width of a few micrometers in a material that is genuine. From then on, the assembly was put in an oxidizing atmosphere in a furnace, where the changes in weight were measured

It was simple to connect the findings to theoretical predictions. It is obvious that microcracks in the matrix in a gasoline environment are common, and evacuation of B_2_O_3_ from a crack is mostly limited by convection around the test. The flow velocity with the oxide is based on the surface associated with the B4C coating that has spread out along with the break. The matrix fracture breadth, plus the gasoline velocity, impacts the rate of volatilization. The environmental factors for self-healing could be inferred by comparing both of these flow prices. When compared to active oxidation, the passive oxidation domain of ecological conditions in a composite is far bigger than that previously observed for garbage [181,182,183,184,185,186,187].

The oxidizing gas flow in a very large fracture is significant, including an important outflow of gaseous hydroxides, not surprisingly for the technical stress of a fractured composite. The rate of volatilization accelerates, rendering self-healing impossible as a result. When a broken composite is mechanically packed, the flow of oxidizing gas within the break increases, causing an outpouring of hydroxides. The price of volatilization accelerates, making self-healing impossible.

### 3.5. Applications

On the other hand, hard solids are sophisticated materials, and composites are required for challenging applications. To extend the lifetime and safeguard engineering buildings, spacecraft, and vehicles, self-healing characteristics must eventually be incorporated into these materials. SiC matrix composites might be employed in several applications with extreme operating conditions, such as jet engines, cogeneration gas turbines, and nuclear fusion reactors.

### 3.6. Summary

Composites with the ceramic matrix are strong components if fiber/matrix bonding is precisely managed throughout the entire production procedure. However, if maintained in a container that is sealed during usage, all substances are susceptible to oxidation. Other components must take in air, preventing it from distributing to materials that can be less resistant. These elements operate as a chemical fuse to avoid oxidation concerning low-resisting materials. No matter the materials used or the heat and climate, self-healing happens when a phase is condensed, shutting any fractures or pores. Oxide corks block the oxygen diffusion route network, preventing air from migrating deeply into the composite. This diffusion is considerably paid off because air usage occurs simultaneously through the oxidation of matrix elements. The air permeability, thermal and security, chemical thermo-mechanical properties, and viscosity of a liquid can all suffer from the oxide structure, due to diffusing from its source and filling any gaps.

At low temperatures, the fluid settling is formed, meaning that the application may end in poor thermal and chemical security at greater performing temperatures. Therefore, a composition having a different oxide combination is developed, which possesses stable oxides within the required range. The structure of the created oxide that is protective is still being researched so to boost its security under corrosive circumstances such as oxidation and limit the oxygen permitted. The self-healing mechanisms of existing items and work that is healthier are well-defined in commercial applications.

## 4. Self-Healing Polymer Composites

Polymers are frequently found in commercial engineering for a variety of reasons, including low weight, high processability, and chemical consistency in a variety of settings. The durability that is the long-term security of polymeric materials found in structural applications, on the one hand, has baffled scientists and engineers [188,189,190]. On the other hand, if polymeric components are exposed to harsh conditions, they could crack/break quickly. Micro-cracking is one of the most dangerous deteriorations that can occur during operation, causing composites to collapse catastrophically and buildings that be drastically reduced. Considering their activities, polymers are divided into two kinds when heated: thermosets and thermoplastics. The self-healing of microcracks in thermoplastic polymers has yet to be shown. The procedures open to thermosetting polymers, which will be addressed at length below, are not suited for thermoplastics because of the limitation of the substance’s nature. However, a few pilot research reports have been performed. Artificial procedures (such as applying heating and solvent) enable the curing of cracks, for instance, through welding. Crack healing was studied in several samples of poly (methyl methacrylate) (PMMA) with varying weights, which are molecular levels of copolymerization. Fracture recovery ended up being achieved by heating the examples above the cup change heat while using modest pressure. It is observed that maximum resistance was restored during short-term loading studies.

### 4.1. Planning of Microcapsules Full of Epoxy Representative

**Figure 17:**
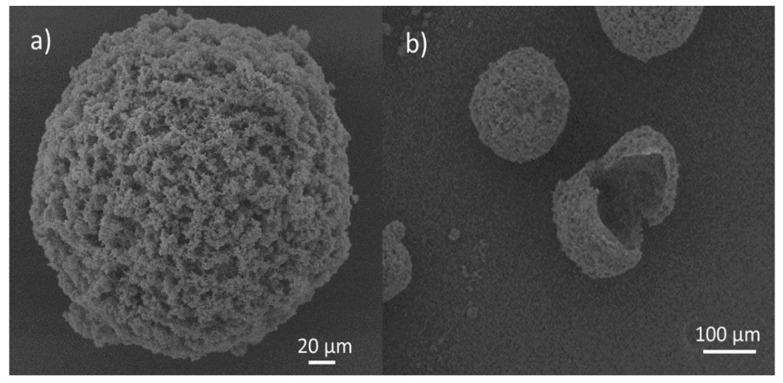
(**a**) SEM image of urea-formaldehyde epoxy capsule (**b**) Cross-section of ruptured capsule shell [193].

Cured epoxy (in other words, epoxy resin bisphenol-A) was microencapsulated utilizing a two-step urea-formaldehyde solution as the wall material. The standard procedures are listed below. The pH of the solution was modified to 8.0 utilizing 10% NaOH after combining 50.4 ml of formaldehyde (37% wt.) with 20.0 g of urea. The water-soluble urea pre-polymer formed a one-hour reaction at 70 °C. In contrast, sodium polyacrylate (PAANa) solution of 800 ml (1.5 wt percent, pH = 8.0), 40.0 g of epoxy was mixed with resorcinol (4.0 g), NaCl (4.0 g), and polyvinyl liquor (4.0 g) (PVA, 0.8 g). Mechanical churning at 16,000 rpm was used to form an oil-in-water (O/W) emulsion. Second, to avoid the methylol urea pre-polymer from interfering with the previous solution method, the epoxy emulsion was linked with the methylol urea pre-polymer. Following that, a 10 percent HCl drip was given to the system regularly. Additionally, the matter was heated (pH 4.0). The device was slowly heated to 70 °C after the pH reached 2.8–3.0. The pH of the system was modified to 1.5–2.0 after 1 hour of heating [191,192]. The product was then neutralized (pH 7) using NaOH, cooled, filtered, and dried, supplying epoxy encapsulated curing in urea-formaldehyde resin (Figure 17). Preparing the latent hardener was fairly straightforward. In 50 ml of methanol, CuBr_2_ (11.2 g) was dissolved (16.4 g). The fluid is diluted by the addition of 150 ml of acetone after mixing for a time, resulting in precipitation. The precipitate is filtered, cleaned, and dried (i.e., CuBr_2_ and 2-methylimidazole complexes). The production price is just about 97.1 percent.

### 4.2. Characterisation of Encapsulated Epoxy

**Figure 18:**
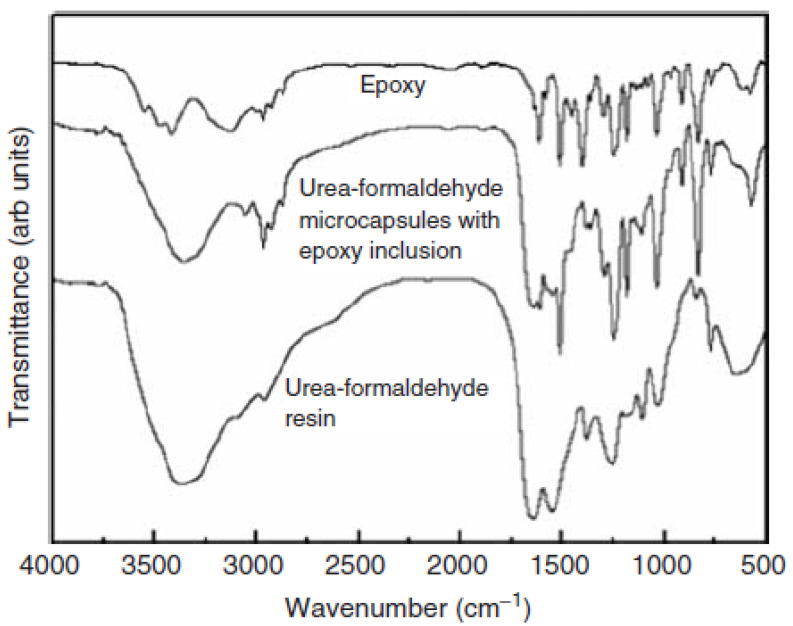
FTIR spectra of epoxy, epoxy filled in urea–formaldehyde capsules, and resin [6].

Since its inception in the 1950s, microencapsulation technology has enhanced rapidly [194]. While many studies have employed urea–formaldehyde resin as a wall content, epoxy-containing microcapsules have yet to be described. As previously indicated, formaldehyde and urea were pre-polymerized within our lab before in situ condensation was used to help make microcapsules. The influence of pH on the services and products was determined to be important in the response process. Exorbitant reaction and basicity time triggered the deposition of white particles if the pre-polymer of methyl urea started to polymerize as water-soluble methylol urea pre-polymer under alkaline conditions. The clear presence of epoxy emulsion impeded the curing of pre-polymers which may be utilized while the wall material is finalized. The removal reaction between methylol urea molecules was generated by the catalysis of alkali or acid after interacting with epoxy emulsion. As a result, low relative molecular mass linear or branched prepolymers were coupled with methylene, methylene ether, and cyclic connectors between urea units, developing over time into water-insoluble polymer connections and depositing into oil–solar networks of epoxy droplets to generate microencapsulated epoxy. It is worth mentioning that urea and methylene would not react when condensation polymerization had been carried out in an alkaline environment, resulting in a dimethylene ether bond rather than methylene linkage. This reduces the system’s performance and, consequently, the product’s cross-linking density, which can be detrimental to the microencapsulated wall energy. The outcome of methylol urea polycondensation was predominantly limited by methylene bonds, favoring chain elongation and the creation of highly cross-linked structures in an acidic environment. The FTIR spectrum of repaired epoxy, epoxy-filled microcapsules, and urea-formaldehyde resin synthesized under the same conditions as the microcapsules revealed a gradual decrement in pH value, as shown in Figure 18. This figure indicating high acidity during the first phase will result in a reaction price that may not be regulated. In the spectra of microcapsules, hefty absorptions at 3300–3500 cm^−1^ wavelength, which suggest the stretching modes of urea–formaldehyde resin-OH and –NH, are noticeable [195,196,197,198]. The spectra associated with microcapsules also show other urea–formaldehyde characteristic peaks. Amine bands (at 1600–630 cm^−1^ and 1530–600 cm^−1^) and epoxy peaks (such as terminal epoxide group bands at 914 cm^−1^ and CH_2_ at 2873 and 2929 cm^−1^) are examples. The urea-formaldehyde microcapsules contain epoxy, as predicted.

### 4.3. Mechanical Characteristics

#### 4.3.1. Tensile Characteristics of Self-Healing Epoxy

**Figure 19:**
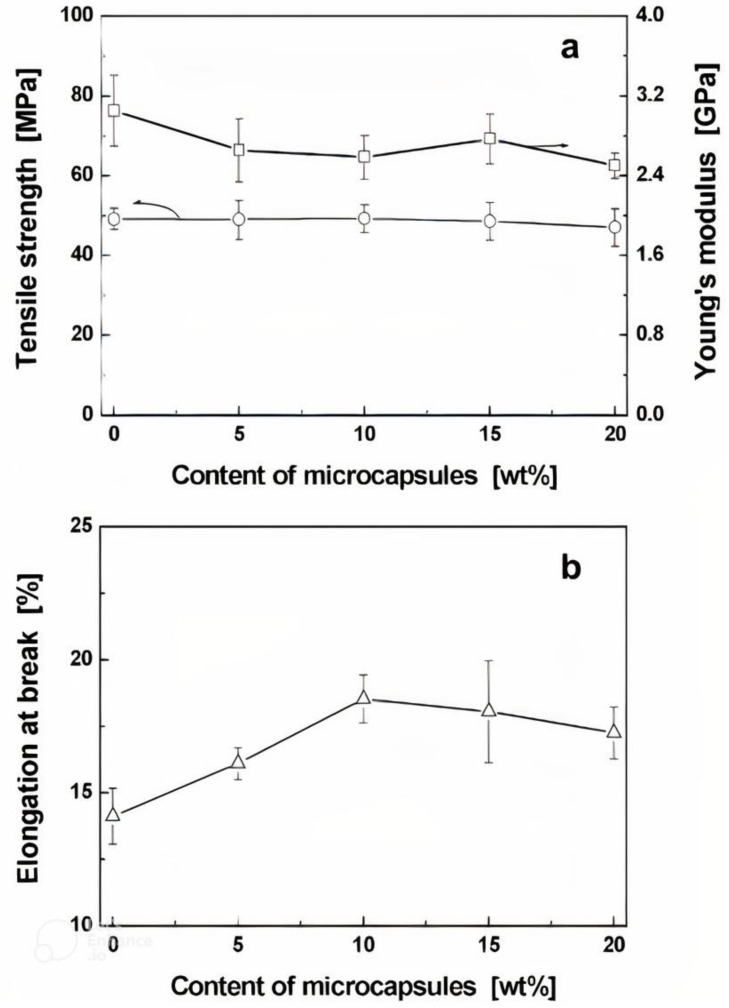
Variation in Tensile strength (**a**) and Elongation at break (**b**), as the microcapsules content increases [202].

As epoxy is microencapsulated, the latent curing agent has to fill the matrix of composites; it is critical to comprehend how they affect the matrix. The matrix’s mechanical efficiency is at its most fundamental level. As a consequence, for a specific CuBr2 (2-MeIm)4 material, the tensile parameters of this epoxy discovered in the self-repair device were calibrated as a function of microcapsule content. The compound tensile power of the microcapsule structure remained very nearly constant, having an improvement, as shown in Figure 19a. It differs from the findings of [199], which unearthed that implanted microcapsules paid off the strength of the epoxy. In general, incorporating rigid or plastic particles into polymers diminishes the matrix’s tensile energy greatly [200]. Nevertheless, if the relationship between the fillers and the matrix is sufficiently strong or the particle size is bigger than the nanometer ranges [201], one will find counterexamples in inorganic particle composites. The urea–formaldehyde resin used in the microcapsule shell works with epoxy resin, as illustrated in Figure 19a, and a good interfacial connection is achieved during curing. The microcapsules can withstand the load sent via contact, unlike soft plastic. This describes the reason the microcapsule material has an impact on tensile strength. The research is aforementioned aided by the pattern of Young’s modulus fluctuation (Figure 19a). In addition, at a filler degree of around 20 percent, the decrease in the epoxy specimen module is not considerable, regardless of the tightness of the microcapsules varying between difficult particles and soft rubber. The microcapsules result in interfacial deformation that is viscoelastic matrix-yielding, leading to a rise in failure strain (Figure 19b). As a result of the uneven distribution of strongly loaded specimens, stress concentration in parts of the specimen might have occurred, resulting in a reduction in elongation at break.

#### 4.3.2. Epoxy with Self-Healing Fracture Toughness

**Figure 20:**
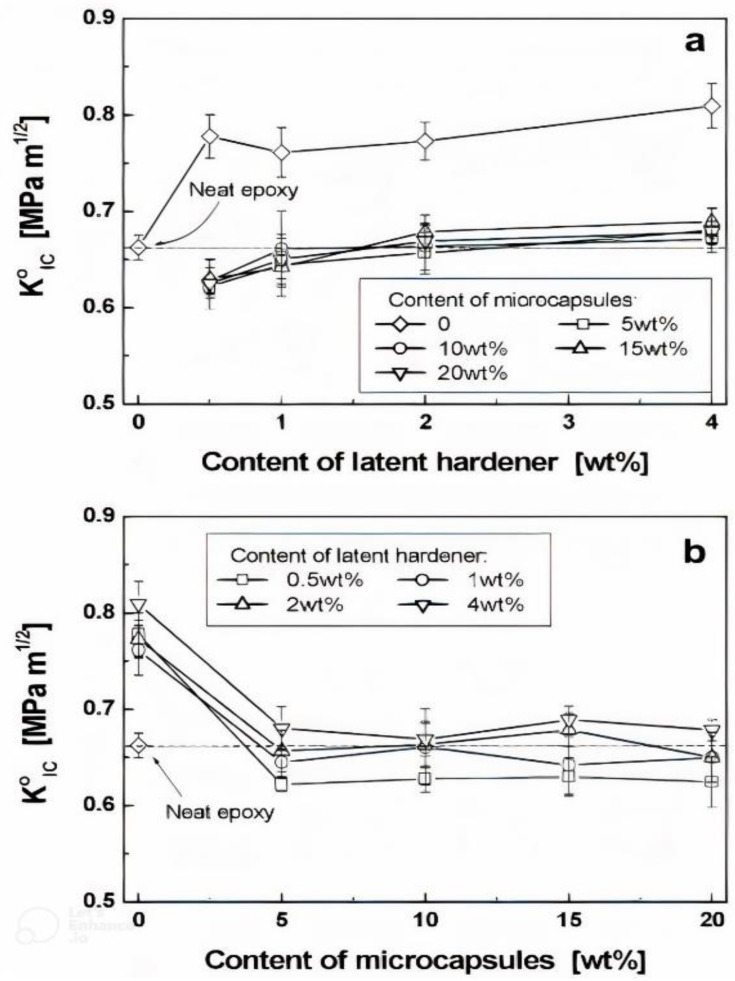
Variation in Fracture toughness, as the latent hardener content (**a**,**b**) microcapsules content increases [202].

The component preceding the fracture strength of the epoxy healing agent is investigated right here (Figure 20). CuBr_2_ (2-MeIm)4 latent hardener incorporation continues to toughen as much as 4% of healed epoxy, as shown in Figure 20a. The plasticizer part is anticipated to be offered by the latent curing dissolved in epoxy. If both microcapsules and latent curing are used, the product’s break capacity is significantly lower than that of natural epoxy. Generally, microcapsules do not prevent fractures from spreading or results in energy absorption. Concerning the positive results of the latent hardener, there is also a negative effect on the fracture resistance of the blends similar to the whole epoxy, (Figure 20). Analyzing the results, it is observed that a two-component self-healing system might not significantly alter the mechanical properties of the epoxy but it leads to a bit-increase in toughness. Instead, in a few circumstances, it plays a part in the qualification of toughening.

#### 4.3.3. Fracture Resistance of Repaired Epoxy

**Figure 21:**
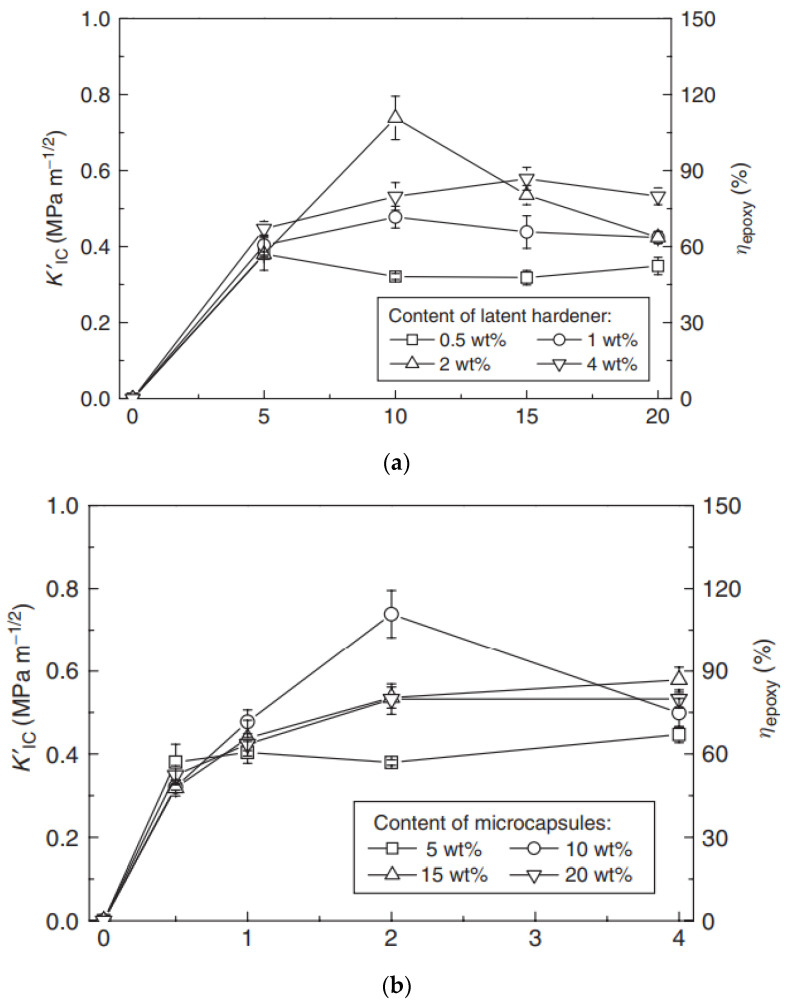
Self-healing ability of epoxy-based on different contents: (**a**) latent hardener and (**b**) microcapsules [6].

The polymerization of the crack-released healing agent is required for crack recovery in polymer composites, which the latent hardener can facilitate. To analyze the break energy of these specimens before and after curing, a few materials with various microcapsule–latent hardener ratios must be created (Figure 21) since an exact percentage of the elements may not be predicted because of this specific situation. Figure 21a depicts the fracture strength of the restored specimen as a function of time. The increased latent hardener could perhaps not guarantee the best cross-linking degree. Hence, the repair’s impact should be restricted suitably. These devices, with 15 and 20 percent microcapsules, lowered the regions of cracked planes that may evenly be repaired. The number of latent hardeners that reach the epoxy released is insufficient, resulting in lower repair efficiency. It would be best to remember that the recovery performance that is greatest shown above is 111 percent, showing that the healed sample’s fracture severity is more than the virgin samples. To comprehend this, epoxy break strength was solely treated with CuBr_2_ (2MeIm)_4_ at a concentration of 5 wt %. The 0.81 MPa m ^1/2^ includes a regularity 1.23 times that of tetraethylenepentamine-curing epoxy, which is cumbersome. The bonding agent then repaired the fractures and supplied the impacted areas with greater break toughness.

**Figure 22:**
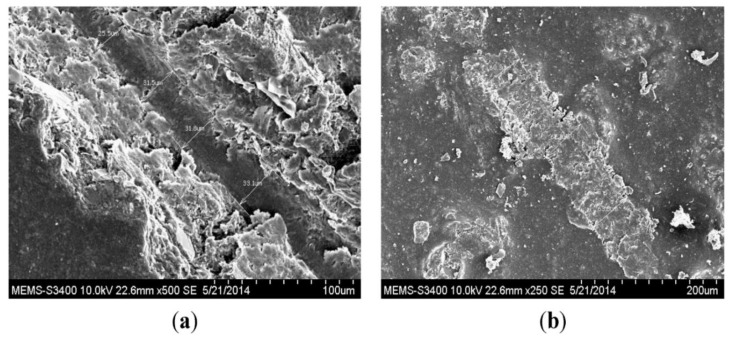
SEM pictures of (**a**) fractured virgin specimen (**b**) fractured healed specimen [203].

The microcapsules were weakened after failing the initial single-edge bending (SENB) test, and the recovery agent flowed away, leaving ring-like concaves (Figure 22a). The specimen is characterized by more advanced fracture patterns (Figure 22b) after healing and moving to the second SENB evaluation. Crack propagation by the healing agent is seen within the thin layer with the shattered surface.

## 5. Fiber-Reinforced Epoxy Self-Healing Composites

**Figure 23:**
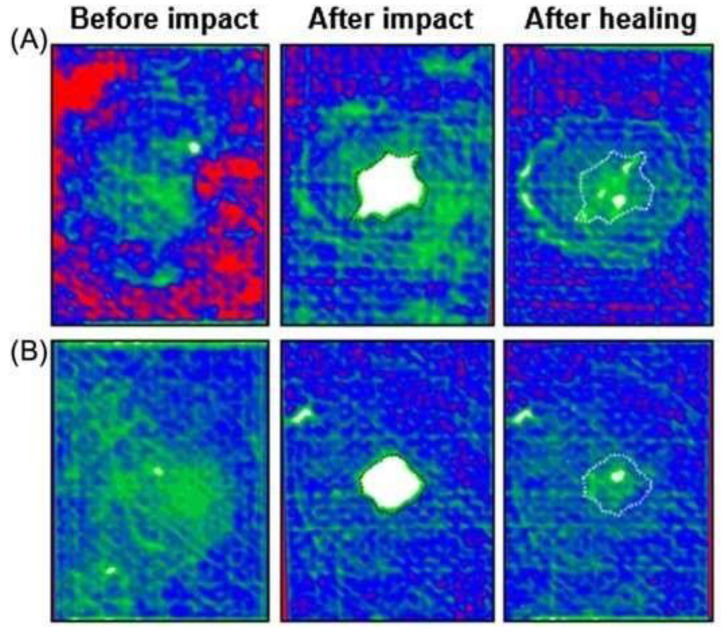
Damage induced due to impact before and after the test of (**A**) CFRP with nylon, (**B**) CFRP with MWCNT and nylon. The left side images show C-scans of the unaffected region before the test while the low velocity impact after the test can be seen in the images presented in the middle. The damage in specimens is due to delamination, as shown in the right side images of both (**A**) and (**B**) [206].

Self-healing of polymer matrix composites is a technique that is unique in self-repairing matrix cracking and delamination. Self-healing polymers might help damaged composites retrieve their mechanical properties, enabling them to fix partially or entirely. Self-healing chemical substances help restore damaged products’ technical properties, making elements that are structurally durable and stable with a longer lifespan [204]. Mendable polymers may help a material restore technical, physical, and tensile properties, such as area smoothness, break power, and buffer properties. Healing occurs whenever a break propagates over the vessel and cracks occur, enabling the liquid recovery agent to move to the break orifice by capillary activity. Mechanical stresses and ultraviolet radiation impact the strength and durability of commonly used polymeric composites [205]. Repetitive loading causes microcracks to occur in the composites. To avoid this, self-healing mechanisms are being found in the introduction of built-in polymeric composites that can significantly mend microcracks and, therefore, recover load-bearing capacity after a fracture. The figure below shows the impact characteristics before and after the impact test, followed by the self-healing role. Figure 23A and Figure 23B show carbon fiber-reinforced polymer (CFRP) with nylon fibers assisted and CFRP with both nylon and MWCNTs, respectively. From these figures, it is observed that initially few regions were not filled with the healing agent at the initiation of the crack and finally, all the cracks were filled with healing agents, and cracks were closed which indicates self-healing was done successfully.

It can be observed that almost all the regions were filled with healing agents and strong nylon connections were seen all over the structures [206].

Researchers have reported the effectiveness of a successful repair of polymer composites employing the method of the computerized release of an agent. This is certainly healing, from either microcapsules or hollow fibers. This system of self-healing has already been enhanced by the growth of mesoscale and microscale vascular, exploring a consistent self-repair function [207]. Microencapsulation with two split healing agent products was developed: (1) urea-formaldehyde enclosed microcapsules with epoxy as the binder material, (2) a hardener molecule that is 2-methylimidazole.

The microcapsules are released due to the initiation and progress of fractures in epoxy matrix-based polymer composites, such as pre-embedded epoxy with a hardener that is pre-dissolved inside the composite. Mending was assisted by a chemical reaction employing the catalyst surrounded by the hardener after the discharge of healing liquids through the microcapsules. This technique helps to restore the location that is damaged.

### 5.1. Epoxy Resin

**Figure 24:**
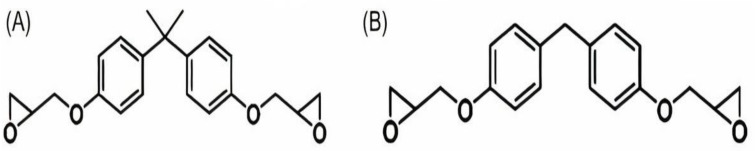
(**A**) Epon 828-bisphenol A (**B**) Epon 862-bisphenol F [208].

A molecule with numerous epoxide groups is called epoxy resin. The properties of epoxy resin are given in Table 3. It improves adhesive, chemical, physical, and mechanical properties when hardened with a proper curing agent such as an aliphatic amine. Figure 24 depicts the structure of epoxy resins.

Glycidyl, non-glycidyl, and epoxy are the two most well-known forms of epoxy resins. The three kinds of glycidyl epoxy resin are as follows: Glycidyl-ether, glycidyl-ester, and glycidyl-amine which are also examples of glycidyl compounds. Nonglycidyl epoxy is an aliphatic or cycloaliphatic epoxy resin. In terms of treating quality and development prices for self-healing composites, epoxy resins may be one of many resin materials best for healing compared to other healing substances. The polymerization period occurs when the epoxy resin binds to the catalyst, allowing the curing agent to mend the break within. The technical qualities of the product are restored by using enough liquid resin as a curing representative to cover the break opening completely. Even though this device requires mending agent channels, and continuous self-healing inside the polymer composite is not possible, the benefit of autonomous self-healing is the absence of external engagement in the treating process [206,207,209].

Epoxy resin is utilized as a matrix in aerospace composites for two reasons: first, it has excellent technical features, and second, it can be produced with low curing temperature and viscosity. The low curing temperature combined with the low viscosity allows for the inclusion of discrete and rather poor microcapsules and their dispersion without premature capsule collapse.

#### Self-Healing Epoxy-Based Polymer

Self-healing epoxy-based polymers are recognized for their specific high strength and modulus, as given in Table 4. Fundamental energy is measured using the strength-to-density ratio. The specific modulus is described by its modulus and density ratio. A product with high energy and specific material modulus has high performance and is lightweight. Glass fiber-based composites have low modulus with high density and a fundamental module of glass dietary fiber resin matrix that is much smaller than metals.

The use of thermoplastic additives in epoxy composites based on E-glass fiber was examined, and the observed healing agent dissolved in epoxy resin forms the single-phase polymer matrix [208,210]. If the epoxy is mendable, it is heated up at elevated temperatures and self-healing occurs/begins.

The thermoplastic transfers through the epoxy resin to solidify and fixes the crack [Yin and co-workers, 2007]. Its purpose was to explore the effects of low-speed results on polymer composites reinforced with cup fiber and implanted with microcapsules. The scientists utilized epoxy-loaded microcapsules as a hardener. In an epoxy-based hardener system, epoxy and hardener were encapsulated separately in the matrix, and when a crack occurs the capsules rupture and flow through the crack regions and repair the damaged portion.

### 5.2. Applications of Self-Healing Composites in the Aerospace Sectors

In this section, the applications of self-healing polymer, metal, and ceramic matrix composites in manufacturing various aircraft parts are discussed in detail. In general, self-healing PMCs are being widely used in various load-carrying structural parts and inlet propeller blades, where high strength-to-weight ratio, corrosion-resistance, and low temperatures are of primary importance. However, self-healing MMCs are being widely used where medium temperatures are of primary importance, while self-healing CMCs are being widely used in engine turbine blades and nozzle design, etc., where high temperatures are of primary importance.

#### 5.2.1. Aircraft Primary and Secondary Structural Load-Bearing Applications

Fiber-reinforced epoxy composites are being widely used in aircraft primary structural load-bearing applications (fuselage, wing, etc.). Fiber-reinforced epoxy composite with 50 wt. % material was used to fabricate civil aircraft. Fiber-strengthened epoxy composites have recently turned out to be suitable for harm or break accessibility, fulfilling certain demands for safe and reliable plane construction.

In addition, since damage to polymer composites is tough to spot and quantify, further vigilance is vital. An extremely effective way of repairing influences the production of safe and lightweight composite fuselages using self-healing polymer composite. The impact strength of carbon fiber-reinforced epoxy composite was examined using self-healing polymer composites [210].

This research discusses the composites’ ability to self-heal as well as their flexural and compressive energy. The flexural energy of 16-ply composite with reinforcement of hollow fibers used in aerospace was reported. The recovery after the quasi-static compression test is measured, is an exceptional amount of 97%. Also, the same composite after the impact test under the compression test reported 92% recovery by self-healing. Both findings indicated that self-healing polymer composites might be used in aircraft primary structural load-bearing applications.

Composites with self-healing fiber-reinforced polymer are becoming more frequent in aircraft elements than architectural applications. The composite has high resilience to cracking since it can fix the crack before break propagation contributes to failure. Hong et al. [205] reported the self-healing procedure for the development of mechanical properties in polymer composites to check for the suitability of airplane structures. The composite was made by using diglycidyl epoxy, it was mixed with bisphenol-A and then implanted with urea-formaldehyde microcapsules. The composite’s elastic modulus was discovered to be notably decreased due to the catalyst addition toward the epoxy matrix. However, the moment the composite had healed, an improvement in mechanical properties was seen. According to research, self-healing polymer composites can be utilized in future aircraft and automotive applications. Additional evaluation using composites of aerospace-grade glass fiber polymers must be completed.

#### 5.2.2. Engine Applications

Conventional ceramic composites are being used in jet engines due to their high thermal resistance; there is also a drawback of CMCs, as they are sensitive to impact damage and brittle fracture which makes them impossible to use in turbine blades, wings, fuselages, etc., which are exposed to impact [211]. Additionally, with conventional CMCs, the engine efficiency has been limited due to their melting temperature, which prevents the engine manufacturers from being able to increase working temperatures. This drawback made the use of ceramic composites limited in the aerospace sector and several studies have been carried out to use CMCs in aerospace for fixed and mobile jet engine components [212].

Replacing conventional CMCs with self-healing composites in engine parts has been executed with the help of a multilayered boron-containing matrix. Under relatively low temperatures, the oxidation behavior of SiC (fibers)/Si-B-C (matrix) was studied and it was observed that in self-healing ceramic composites, the healing action is due to the formation of boron oxide (B_2_O_3_) which can seal the cracks in the matrix [146].

CMCs made of 2D-C/(SiC-[B-C]) materials have been tested at high temperatures such as 1000 °C, 1200 °C, and 1400 °C, which were found in the aircraft combustion chamber. Self-healing was attained when boron compounds and boron silicon glass compounds were oxidized, which led to the flow of phases into the matrix cracks and sealing them [213]. From the three-point bending test and tensile test, strength has been increased for these ceramic composites with the temperature. Self-healing has also been triggered and these CMCs can be suited for aircraft combustion chambers.

Boron usage in aerospace was further investigated and it was found that the addition of pyrocarbon to the boron-doped matrix increases the corrosion resistance and self-healing properties. The multi-layered matrix containing B-doped pyrocarbon and B_4_C and SiC layers along with SiC-SiC fiber reinforcements was studied and it was observed that boron-bearing elements in the interphase layers and in the matrix itself provide self-healing ability by forming B_2_O_3_ under oxidizing environment. This type of composite has excellent corrosion resistance under mechanical loads and temperature resistivity which is suitable for jet engine combustion chambers [213].

#### 5.2.3. Aerostructures

FRCs and epoxy composites have been widely used in airplane structural parts, as they have greater fatigue resistance, because of their capacity to heal micro-cracks and prevent them from extension which leads to failure [214]. A study was conducted to assess the effects of the healing ability of composite materials and mechanical properties to determine their use in aero-structural applications. For this study, a composite made of DGEBA epoxy matrix embedded with DCPD containing urea-formaldehyde microcapsules and observed after the mechanical load, and good recovery of mechanical properties resulted after the healing action [116].

When self-healing epoxy composite embedded with HGF as a healing agent and underwent indentation and a three-point flexural test and results confirmed that there is good strength recovery in the composite after healing. These investigations stated that self-healing HGF polymers have strong potential and can be used for aerostructures [196,215].

Similarly, E-glass-epoxy FRCs can also be considered for aerostructures after the incorporation of E-glass-epoxy plates as a vascular network, parallel to the fiber’s direction. In this section, Lewis acid-catalyzed epoxy was treated as a healing agent and it was observed that the composite has fully recovered its pure mechanical characteristics and understood that self-healing glass epoxy composites can be wise alternatives for fiber-reinforced polymer composites [216,217].

Carbon fiber-reinforced polymer composite has been widely used in aerospace structural applications [8]. A high-pressure molding process has been suggested for manufacturing carbon fabric from carbon fiber-reinforced polymer doped with shape memory polymer with carbon nanotubes. Self-healing behavior was exhibited by reversible crosslinking of polymer that helps to attain the system’s shape memory ability with excellent healing in the matrix cracks after the tension failure [121,218].

#### 5.2.4. Coatings

Coatings are crucial in the aviation sector since they protect the fuselage and aerostructures from deterioration. The coatings are beneficial simply because they self-heal after any break development. Coatings in composite polymers have minor significance than aerospace parts because their damage does not affect the entire strength of structures. An epoxy resin containing microcapsules can be treated as a coating material for steel alloy material and acts as an anti-corrosion coating even after damage occurs. Self-healing coated materials are promising materials in aero-applications as they would recover their protection ability automatically after the damage [219].

In military and civil applications, coatings for metallic alloys such as aluminum, magnesium and titanium are important as they are abundantly used materials and need corrosion protection. Self-healing vanadia coatings for aerospace-grade aluminum and magnesium alloys were found to be satisfactory as they prevent oxygen from penetrating and observed that chromate coatings can be replaced with vanadia coatings, especially in aerospace applications [220,221,222].

### 5.3. Summary

Epoxy-based self-healing composite products have been reported broadly and many concepts are explored to be relevant to polymer composites. This process might be utilized in lots of organizations, including aerospace. They treat healing agents similar to microcapsules and can be vascular networks, which helps the method that enhances self-healing effectively. These composites are used more in the aerospace industry regarding fatigue and impact resistance [223,224]. Their deterioration avoidance purpose at first glance is often increased, which might help with harm information recovery. Self-healing PMCs were used for manufacturing aircraft primary (fuselage, wing, etc.) and secondary structural load-bearing (flaps, ribs, spars, etc.) applications, casings for aero-engines, etc. [9,225,226,227]. However, further studies related to self-healing PMCs are required to investigate the effects of size and healing content on the performance of self-healing coating for various applications [228,229,230]. Self-healing MMCs are being widely used for compressor blades of aero-engines, anticorrosion coatings, landing gear applications, etc. [231]. Self-healing CMCs are being widely used for aeroengine applications, especially for manufacturing combustion chambers, turbine blades, nozzle inner and outer structures, etc.

## 6. Conclusions

Many issues such as wetting improvement, avoiding oxidation, maintaining enough capillary pressure, and repairing microscopic damage with no loss of strength are all concerned before designing self-healing metallic materials. When applying self-healing metallic materials to biological applications, structural health management (SHM) provides information on the damage, such as a biological, and neurological system, and information on the type, location, and extent of this damage. New self-healing mechanisms must be developed for low melting temperature alloy fibers such as tin-based alloys/solders, aluminum alloys, and zinc-based alloys. Precipitation regeneration and consumption have been utilized to demonstrate nanovoids and micro-cracks in aluminum alloys, nickel-based alloys, and steels that self-heal.

Taking proper control of fibers or matrices during the fabrication procedure produces strong ceramic matrix composites. Chemicals must be stored in air-sealed containers to prevent oxidation and special care must be taken for other components without exposure to air frequently; these components act as a chemical fuse to stop oxidation. Self-healing always happens in the condensation period, regardless of temperature and climate. Oxygen diffusion occurs in the matrix through crack-forming and the oxygen permeability effects, thermal, chemical, and thermo-mechanical properties.

However, sealing liquid developed at low temperatures has poor thermal and chemical resistance, which creates oxidation but in a limited range at high temperatures; research on this problem is being conducted to prevent a corrosive environment. Self-healing systems for contemporary materials are well-established, together with safe working areas of environmental conditions utilized in commercial applications.

Since the self-healing of polymers and their composites is in its starting stage, complex research is being carried out on new polymers with inherent break-repair capability or combining existing materials with original recovery systems. Curing agent approaches could be useful within the industrialization process. Products produced from these materials can be smart and have a longer service life, causing less trash to be discarded. For example, [207] developed self-healing polymer vascular networks that can cure multiple fractures.

Polymer composites built with epoxy have very prominent applications in the aviation industry, having good resistance to impact and fatigue. Their corrosion avoidance function could be increased, providing additional assistance with damage recovery.

## Figures and Tables

**Table 1:** Factors to be considered while developing microcapsule embedded matrix. [10,11,12,13].

Agents	Agents Functioning
**Microcapsule**	The healing must be chemically inactive to the polymer shell; the capsule must have a long shelf life. It must be compatible with the dispersed polymer region. The shell walls must be weak to allow for rupture. The catalyst must be close to the capsule. The matrix and the capsule must have a high interfacial attraction.
**Monomer Polymerization**	Less volatile for the required time to complete polymerization. No cure-induced shrinkage and stress relaxation. Polymerization at atmospheric temperature
**Catalysts**	Dissolves in monomer, clustering does not occur in polymer matrix
**Coatings**	Microcapsules have a strong influence on the physical and chemical characteristics of the matrix; Microcapsule’s size will be smaller than the thickness of the coating, and clustering does not occur in the matrix
**Healing**	Low economy, must be quicker, multiple cycles

**Table 2:** Healing performance of capsule-based self-healing materials [86].

Mechanism	Healing Percentage (%)	Time for Healing (hrs)	Conditions for Healing	Matrix	Reference
DCPD + Grubbs	75–100	10–48	Room temperature	Epoxy	[10]
DCPD + Grubbs	30	24	Room temperature	Epoxy vinyl ester	[10]
DCPD + Grubbs	70–100	48	Room temperature	Epoxy + CFRC	[10]
DCPD + WCl_6_	20–64	24	25–50 °C	Epoxy	[13]
ENB + Grubbs	45–80	48	Room temperature- 80 °C	Epoxy	[14]
ENB/DCPD+ Grubbs	85	48	Room Temperature	Epoxy	[14]
ENB + Hoveyda Grubbs	95	2	170 °C	Epoxy	[14]
HOPDMS and PDES	20–24	24	50 °C	Epoxy vinyl ester	[23]
HOPDMS and PDES	100	48	150 °C	Epoxy +FRC	[23]
Epoxy and Solvent	85–100	24	Room Temperature	Epoxy	[46]
Epoxy and Solvent + Scandium triflate	More than 80	48	80 °C	Epoxy	[50]
Epoxy + CuBr (2) (2-Melm)4	111	1.5	130 °C-180 °C	Epoxy	[64]
Epoxy + Mercaptan	104	24	20 °C	Epoxy	[64]
Epoxy + MBM tetrathol	120	5 days	25 °C	Epoxy	[75]
Epoxy + antimony pentafluoride	70	20 sec	Room temperature at 0.2 MPa	Epoxy	[75]

**Table 3:** Characteristics of epoxy resin [22,189].

Property	Value
Viscosity (cP)	12,000–13,000
Density (g/cm^3^)	1.16
Tensile strength (MPa)	73
Elongation(%)	4
Flexural strength (MPa)	60
Heat distortion temperature(°C)	100”
“Property	Value
Viscosity (cP)	12,000–13,000
Density (g/cm^3^)	1.16
Tensile strength (MPa)	73
Elongation(%)	4
Flexural strength (MPa)	60
Heat distortion temperature(°C)	100”

**Table 4:** Properties of materials with different structures [24,210].

Material Type	Density	Tensile Strength (GPa)	Specific Strength (GPa)	Young’s Modulus (GPa)	Specific Modulus (GPa)
Epoxy-carbon fiber II composite	1.45	1.50	10.3 × 10^6^	1.4 × 10^2^	9.7 × 10^8^
Epoxy-carbon fiber I composite	1.6	1.07	6.7 × 10^6^	2.4 × 10^2^	15 × 10^8^
Epoxy-organic fiber composite	1.4	1.40	1.0 × 10^6^	0.8 × 10^2^	5.7 × 10^8^
Epoxy-boron fiber composite	2.1	1.38	6.6 × 10^6^	2.1 × 10^2^	10 × 10^8^
Epoxy-glass fiber composite	2.0	1.06	5.3 × 10^6^	0.4 × 10^2^	2.0 × 10^8^
Aluminum- boron fiber	2.65	1.0	3.8 × 10^6^	2.0 × 10^2^	7.5 × 10^8^
Steel	7.8	1.03	1.3 × 10^6^	2.1 × 10^2^	2.7 × 10^8^
Titanium alloy	4.5	0.96	2.1 × 10^6^	1.14 × 10^2^	2.5 × 10^8^
Aluminum alloy	2.8	0.47	1.7 × 10^6^	0.75 × 10^2^	2.6 × 10^8^

## Data Availability

Data will be provided upon request.
